# Identifying a New Social Intervention Model of Panic Buying Under Sudden Epidemic

**DOI:** 10.3389/fpubh.2022.842904

**Published:** 2022-03-11

**Authors:** Peihua Fu, Bailu Jing, Tinggui Chen, Jianjun Yang, Guodong Cong

**Affiliations:** ^1^School of Management and Electronic Business, Zhejiang Gongshang University, Hangzhou, China; ^2^School of Statistics and Mathematics, Zhejiang Gongshang University, Hangzhou, China; ^3^Department of Computer Science and Information Systems, University of North Georgia, Dahlonega, GA, United States; ^4^School of Tourism and Urban-Rural Planning, Zhejiang Gongshang University, Hangzhou, China

**Keywords:** panic buying, social intervention, behavioral decision-making, the COVID-19 pandemic, sudden epidemic

## Abstract

COVID-19 that broke out at the end of 2019 continues to spread globally, with frequent occurrence of variant disease strains, thus epidemic prevention and control become a kind of routine job. At present, due to the prevention and control measures such as maintaining social distance and community blockades, there is a boom in material purchases in many places, which not only seriously endangers social order and public environmental safety, but also easily leads to the interruption of the supply chain and the shortage of social materials. This article aims to study the intervention methods to curb the spread and spread of panic buying behavior. Firstly, through crawler technology and LDA (Latent Dirichlet Allocation) topic model, this article analyzes the intervention measures taken by various social forces in China to curb the spread of panic buying, and summarizes the multi-channel intervention measures including online and offline forms. Secondly, through the multi-Agent Monte Carlo method, the targeted intervention mechanism is supplemented in each propagation link of the panic buying propagation model, and a new social intervention model of panic buying under sudden epidemic is constructed. Then, through MATLAB modeling and simulation, the main factors affecting panic buying intervention are discussed. The simulation results show that: (1) The single plan with the best intervention effect is the supply monitoring. While the official response can play an immediate inhibitory effect, but it is affected by credibility and timeliness. The intervention effect of psychological counseling is limited, and it generally needs to be used in combination with other measures. (2) The combination strategy with the best intervention effect is “supply monitoring + official response + psychological counseling,” and the worst is “information review and guidance + psychological counseling.” Supply monitoring is a key measure to curb panic buying. At the same time, “information review and guidance” will have a certain counter-effect in the combined strategy. Finally, the effectiveness and universality of the proposed model are verified by examples of China and Britain.

## Introduction

The COVID-19 pandemic that broke out at the end of 2019 has had a serious impact on human society, as it turned out, many cities have seen irrational panic buying incidents. At present, with the emergence of various mutant disease strains around the world and the gradual opening of entry and exit, the prevention and control situation is still severe, and panic buying incidents still exist. For example, in June 2021, the sudden increase of 24 community cases in the New South Wales region of Australia led to a 2-week period of martial law in Sydney, followed by panic buying of toilet article (1). Frantic shoppers broke out in fights over toilet article, and social media was full of pictures of empty supermarkets. The Australian prime minister had to issue a public statement to curb panic buying as a result of mass buying and hoarding by some people, making it difficult for ordinary people to get supplies. Such phenomena have also occurred in United States (2) and Japan (3). This kind of panic buying not only seriously endangers social order and public environmental safety, but also easily causes a shortage of social materials. Therefore, studying effective social intervention measures and using social forces at all levels to curb the spread of panic buying have important theoretical and practical significance.

According to the definition of Oxford Dictionary (2020), panic buying is “The action of buying large quantities of a particular product or commodity due to sudden fears of a forthcoming shortage or price rise (4).” Scholars also have similar views. For instance, Arafat et al. (5) believed that panic buying might refer to the phenomenon of a recent increase in business of one or more essential goods in excess of regular need promoted by advertisement, usually a disaster or an outbreak resulting in an imbalance between supply and demand. At present, the research on this phenomenon mostly focuses on the analysis of causes. Scholars have discussed from the perspectives of commodity supply and demand (6), panic mood (7), social media (8), and so on. However, how to deal with or reduce the occurrence of panic buying incidents is rarely mentioned, and this is the research content of social intervention measures. Generally speaking, social intervention measures is a social coping mechanism, which refer to social forces such as governments, private institutions, and social organizations that borrow various measures before and after panic buying events to help people solve actual needs, restore psychological balance, and alleviate panic buying behavior. With regard to social intervention, scholars have conducted research from the perspectives of the government (9), enterprises (10), and individuals (11), but they mostly focus on other social issues, for example, curb information dissemination (12), reduce social loneliness (13), etc. There are few intervention studies on panic buying, and most of them are qualitative analysis, which lack of quantitative discussion. Quantitative analysis can more flexibly observe the model effect by adjusting the model parameters, that is, the effect of intervention measures.

Taking COVID-19 as the background, this article discusses the panic buying behavior under sudden epidemic. Compared with the past sudden epidemic such as Sara and Ebola virus, the panic buying event under COVID-19 has more timeliness, wider global influence and more prominent research significance. In addition, this article integrates the causes of panic buying and existing social intervention measures, and constructs a social intervention model for panic buying behavior in an emergency. The structure of the article is as follows: Section Literature review is a literature review. Section Sorting out social intervention methods analyzes data about panic buying incidents and related news during China's anti-epidemic period, and combines references to sort out social intervention measures for panic buying. Section Model construction constructs a social intervention model for panic buying under the sudden epidemic. Section Simulation experiment analyzes the intervention effects of different measures on panic buying behavior through simulation experiments. Section Empirical analysis verifies the effectiveness and applicability of the proposed model through two real cases. Section Conclusions summarizes the article and prospects for future work.

## Literature Review

Panic buying is a common group behavior in emergencies such as earthquakes, hurricanes, and epidemics. For example, due to COVID-19, people are rush to buy products such as hand sanitizers, medicines, masks, and food around the world. Usually, the uncertainty of the environment, the induction of panic emotion, and the purchase of products that exceed one's own needs are the common features of panic buying. Collect relevant literature by keyword search on the web of science, read it one by one after coding, and classify it according to different contents, which is mainly divided into two aspects: the causes of panic buying and the intervention mechanism, as shown in Table 1.

**Table 1:** Research directions of panic buying.

**Research direction**	**Specific perspective**	**References**
The causes	The imbalance of commodity supply and demand	(5, 14, 15)
	Individual panic emotions	(16–18)
	The role of online social media	(19–22)
The intervention mechanism	Government perspective: government communication, government prevention and control, etc	(23–26)
	Enterprise perspective: maintain market supply balance, regulate product prices, etc	(17, 27–29)
	Individual perspective: interpersonal intervention, behavioral cognitive therapy, etc	(13, 30–33)
	Comprehensive perspective: combined with the three-dimensional perspective of government, enterprises and individuals	(34–37)

At present, scholars' research on panic buying behavior is mainly focused on its causes. They believe that the imbalance of commodity supply and demand, individual panic emotions and the role of online social media are the three main factors that cause panic buying behavior.

Regarding the research on panic buying caused by the imbalance of commodity supply and demand, some typical literatures are as follows: Arafat et al. (5) collected media reports with “panic buying” as the key word and found through statistical data analysis (14) that the sense of scarcity of products was an important factor leading to panic buying during COVID-19. Wang and Holly (15) took three cities in China as samples and adopted the multivariable Probit model to study, and found that the amount of food people had and the expectation of the possibility of COVID-19 infection were the main factors affecting food hoarding. As for the panic buying triggered by individual panic, the representative literatures are as follows: Keane and Neal (16) pointed out that government policies such as restrictions and lockdowns in the initial phase of the epidemic caused great panic among the public. Prentice et al. (17) pointed out that this panic led to increased levels of anxiety, chronic pain and overbuying. Bacon and Corr (18) conducted a questionnaire survey of British respondents and found that people were experiencing a psychological conflict between the urge to stay safe and the desire to maintain a normal, pleasurable life, while panic buying was one of the ways to improve this psychological conflict. In addition, regarding the role of online social media, it not only amplifies the scarcity of goods, but also promotes the spread of panic, and further aggravates panic buying behavior. For example, Hao et al. (19) used a bivariate probability model to empirically study the impact of online purchasing channels on food hoarding behavior in Urban China using random survey samples. The results showed that because the scarcity of fresh food products on the e-commerce channel was more intuitive, it was more likely to induce panic buying behavior. Naeem (20) studied the role of social media in creating panic. The study showed that the massive real-time data on social media could not only provide comprehensive decision-making basis, but also make consumers more anxious, leading to panic buying or hoarding of products. Zhou (21) pointed out that a large number of unscientific media reports on emergencies, as well as the informal dissemination of information within the group and the infection of panic, coupled with the lag of emergency measures of government departments, would amplify the psychological expected value of individual participation in rush buying, generate a positive driving force for group rush buying behavior and aggravate the group nature of behavior. Fu et al. (22) analyzed the formation and dissemination process of panic buying behavior by integrating internal and external factors such as commodity supply and demand, individual emotion and herd psychology. The result showed that the number of people in social networks and the release time of external information had an important impact on the dissemination of panic buying.

The above literatures show that scholars have launched a multi-angle discussion on the causes of panic buying. For example, they explain the external causes of panic buying from the perspective of commodity supply and demand balance as well as online social media, and explain the internal causes of panic buying from the perspective of individual emotions, highlighting the important influence of demand, inter-individual interaction, and emotion. Although these literature help people better understand the causes of panic buying, they rarely involve the control or intervention of panic buying. In the context of COVID-19, panic buying for certain types of goods in a short period of time may lead to insufficient supply of goods and the occurrence of social stampede, which is more likely to cause group infections. Therefore, how to effectively curb or intervene in group panic buying is an important social issue. At present, scholars analyze social intervention measures mainly from the three perspectives of government, enterprises, and individuals.

The literatures on intervention measures from the government perspective are as follows: Duan et al. (23) pointed out that the government was the responsible subject in the event of public health events, and the way of government intervention could be divided into three parts: government communication, government prevention and control, and government assistance. Government communication refers to the formation of information communication between the government and the public through announcements and other forms after an incident. There are relatively many studies on this part, for instance, Ye (24) pointed out in the study of Internet emergencies that it was of great importance for the government to make use of the advantages of Internet resources to release real information in the first time, gain the right of online discourse, adjust the irrational motivation of the public and guide the development direction of the incidents. Stuart et al. (25) introduced compensation control theory (CCT). By collecting 14 day big data from 24,153 Twitter users in Italy, text analysis and GLMM generalized linear hybrid model were used to explain panic buying during the pandemic. The results showed that effective government announcement could regulate the anxiety perception of the public and prevent panic buying behavior. Lu et al. (26) constructed a two-layer network diffusion model to describe the intervened information about disease dynamics, and conducted a full space simulation to illustrate the trade-off between information disclosure and blockade. The research showed that when people had a high medical cognition level and high public health awareness about virus, the government took priority to the accuracy of information disclosure rather than the speed of disclosure, but irresponsible government tended to delay information disclosure, while risk averse government tended to block information completely.

Research on intervention measures from the perspective of enterprises, including maintaining market supply balance and regulating product prices are as follows: Tsao et al. (27) studied the impact of different levels of supply interruption and panic rate on supplier decision-making and profit, and pointed out that business practice might mitigate the impact of situational factors and personal factors on panic buying by affecting market supply. Stock and Balachander (28) discussed that sellers used scarcity strategy as the best way to transmit their quality signal to uninformed customers, and pointed out that if enterprises increased product prices or failed to replenish inventory during the crisis, it would be regarded as a signal of scarcity, thus aggravating panic buying behavior. Prentice et al. (17) used the scarcity principle, group psychology and infection theory to explore panic buying behavior, and used retailer intervention as the regulation mechanism, combined with structural equation method. The experimental results showed that the regulation effect of retailer intervention varied with product category. Arafat et al. (29) discussed the characteristics of panic buying events in Bangladesh. Through the content analysis of relevant news reports on Google search engine, authors determined five panic buying events in Bangladesh, and discussed the triggering events, responsibility factors, goods obtained by panic buying and preventive measures. Raising awareness, selling goods at a lower price by the government, formulation of the special monitoring team, punishment to maleficent sellers, dissemination of stock status to the general people, assurance of stocks, import from alternative sources, reduced use of goods (onion) rationing while selling from the super shops, publishing circulars in newspapers to raise awareness, and a reduction of import duty were the controlling measures identified by the analysis.

The study of intervention measures from the perspective of individuals mainly considers the impact of social relationships on individuals. Through combing the literatures, scholars have verified the feasibility and effectiveness of social relationship intervention in following aspects: promote physical exercise (30), and reduce social loneliness (13), which are also called the Connecting People Intervention (CPI) (31). For instance, Webber et al. (32) provided CPI health training for 155 people with mental health problems or learning disabilities, and found that the full implementation of CPI could improve mental health problems or learning disabilities. Kar et al. (33) introduced the five zone model of cognitive behavioral therapy (CBT) of panic buying, tried to explore the impact of online group CBT on panic buying, and demonstrated that the structured treatment of CBT (cognitive reconstruction and behavior correction strategy) might play a role in panic buying, even if it was impossible to carry out structured treatment. At least the core skills used in the CBT process (challenging thinking, gathering evidence and preventing maladaptive reactions) could be used to reduce panic buying behavior.

Some other scholars have also conducted intervention research from multiple perspectives. For example, Menon and Varadharajan (34) focused on outlining the possible preventive measures to control panic buying. The strategies were divided into universal prevention strategies, aimed at the entire population, selective prevention strategies that targeted vulnerable sub-sections of the population and indicated prevention for those showing early signs of the condition. Universal prevention strategies covered the role of governmental agencies, retailers, media, and promotion of kinship/resilience among the public. Selective prevention strategies involved identification of individuals prone to PB, monitoring their behavior and specifying purchase limits for commodities, while indicated prevention involves referral to mental health professionals for those with co-morbid anxiety or depression. Arafat et al. (35) aimed to discuss the control measures that could reduce panic buying and pointed out that media played a vital role in controlling the Panic Buying. Promotion of feeling of kinship and encouraging generosity could reduce it from the public end. Creating a bar for buying the necessary goods and subsidiary sales of necessary goods for people with special needs could be another potential strategy. Social sanctions and behavioral measures might have roles and repeated assurance was needed. Rajkumar and Arafat (36) reviewed summarizes the existing research in the variables influencing panic buying and examines its implications for the prevention and control of panic buying. Providing an empirically tested model of panic buying behavior (Group A) or a theoretical model supported by literature (Group B), were retrieved through a literature search. It was found that a wide variety of primary (crisis/disease-related), secondary (psychological, informational and sociopolitical), and tertiary (supply chain-related) factors were significantly associated with panic buying, while a single variable–reflective functioning was identified as protective. Arafat and Kar (37) pointed out the three-level prevention strategy of panic buying. When the primary prevention strategy was implemented, when the stimulation occurred but the panic buying behavior did not occur, consideration should be given to, such as raising awareness of the emergency, repeatedly ensuring the necessary goods inventory and supply, etc; After the panic buying attack, the secondary prevention strategy should be described by sensitive media, regularly updated the inventory status, maintained the supply chain, reduced import taxes, etc; the implementation of the three-level prevention strategy should maintain stable supply, reasonable media coverage and ensure inventory status after the panic buying attack stopped but before the crisis was relieved.

From the above analysis, it can be seen that the current scholars have mainly conducted research on the causes of panic buying. Although they have noticed that panic buying will have serious consequences, following problems still exist in the social intervention: (1) The research objects are not focused enough. The current researches mostly involve the spread of online public opinion or personal health issues. Although some scholars have studied the intervention measures of panic buying, the number of studies is relatively small; (2) the research methods are mostly single perspective research, and lack of quantitative research. Generally speaking, the social intervention mechanism has three perspectives: government, enterprise, and individual, but scholars mostly conduct research from a single perspective. Even if a comprehensive mechanism is formed, it is mostly qualitative discussion, which is difficult to carry out empirical verification. Therefore, studying the comprehensive and quantitative social intervention mechanism of panic buying has important theoretical and practical value. This article analyzes the existing panic buying social intervention measures, and combined with the causes of panic buying, integrates the intervention measures that can be taken by the government, enterprises and individuals, constructs a comprehensive intervention model at first, and then simulates the impact of different intervention measures through simulation experiments. It is found that different intervention measures have different effects. The intervention effect of Supply Monitoring is the best, while the intervention effect of Psychological Counseling is limited. At the same time, the intervention effect of the combination of Supply Monitoring, Official Response and Psychological Counseling is more obvious. Finally, the panic buying examples in China and Britain are selected to verify the model, and it is found that the model proposed in this article has certain universality.

## Sorting Out Social Intervention Methods

Public emergencies have the characteristics of suddenness and urgency, which can easily cause psychological imbalances, and produce anxiety, panic, anger, depression, and other negative emotions, so as to trigger psychological crises and affect people's social behavior. The social intervention measures for panic buying refers to a social response mechanism that the government, private institutions, social organizations, and other social forces use various measures during and after the panic buying event to help people satisfy their actual needs, restore psychological balance, and alleviate panic buying. However, it is short of specific analysis of social intervention measures for panic buying currently. To this end, this section takes China's most authoritative and credible “People's Daily Online” (38) as the data acquisition platform, investigates panic buying in China during the period from January 1, 2020 to April 1, 2021, uses crawler software and data analysis methods to sort out the social intervention measures in relevant news, and organizes them in conjunction with the relevant literatures, and finally forms a list of social intervention measures for panic buying.

### Date Crawling and Preprocessing

The first step is crawling. Web crawler (39) can capture website information through three algorithms: network topology, web content and user access behavior. It can help us obtain a large amount of network data information and lay the foundation for subsequent analysis. Through the professional crawler tool “Octopus” (40), the news information on “People's Daily Online” is crawled with similar words such as “panic buying” and “panic buying,” and crawled the most relevant 1,035 piece of information data.

The second step is data preprocessing. First, delete news that is out of time. In order to ensure that the information of news is based on the COVID-19, according to the “Release Time,” data other than January 1, 2020 to April 1, 2021 is deleted with total of 249 pieces of data, and 786 pieces of data remain. Secondly, delete irrelevant news, as follows: (1) delete news that occurred abroad, such as the United States, Germany, Australia, France, South Korea, Japan, Indonesia, Turkey, and other countries; (2) delete news that is not related to COVID-19 News, such as buying real estate, buying assistant software, double-eleven buying, live streaming, chip buying; (3) delete duplicates. Part of the news is placed on local websites, Weibo and other channels at the same time, which is not conducive to sorting out the intervention measures. Therefore, the information of news with the same title and summary is deleted. Finally, 612 pieces of data were deleted, leaving 174 pieces of data.

### Event Classification

According to the classification of events according to time, it is found that there are mainly four panic buying events in China with a wide range of influence from January 1, 2020 to April 1, 2021, as follows:

On January 30, 2020, in the early stage of COVID-19, supermarkets in some areas were sold out (41). On March 30, 2020, a small number of people rushed to buy grain and oil in Huangshi, Huanggang, and Ezhou in Hubei Province. The reason was that along with the spread of COVID-19 around the world, some food exporters had reported restrictions on exports, and some citizens were worried that food prices would rise and cause panic (42). On August 26, 2020, affected by the flooding and the unstable international food prices, the operation of China's grain market experienced periodic fluctuations, which aroused widespread public concern, resulting in panic buying of rice in some areas (43). On January 5, 2021, Shijiazhuang, Hebei Province added 20 newly confirmed local cases, and the situation of epidemic prevention and control has suddenly intensified. At the same time, some supermarkets in Shijiazhuang occurred panic buying. Foods such as rice, noodles, oil, instant noodles were sold out, and supermarket shelves were emptied (44).

### Date Analyzing

LDA (Latent Dirichlet Allocation) topic model (45) can give the topic of each document in the document set in the form of probability distribution. According to the content of news text, LDA theme model is used to divide all interventions into 5 categories, namely, government prevention and control, timely response, positive guidance, negative reinforcement, and psychological counseling. Among them, government prevention and control and timely response belong to the government's intervention, which are directly intervened by the government. Positive guidance and negative reinforcement belong to the social perspective, which are directly intervened by news media, non-governmental organizations and other social organizations. Psychological counseling belongs to the individual intervention, which is directly intervenes by individuals such as psychologists.

It can be seen from Table 2 that social intervention is mainly based on psychological counseling, timely response and positive guidance, supplemented by government prevention and control, and negative reinforcement. The different types of measures are classified on a monthly basis and correspond to the panic buying event. The results are shown in Supplementary Figure 1.

**Table 2:** Social intervention measures.

**Classification angle**	**Category**	**Classification description**	**Data volume**
Government	Government prevention and control	Implement prevention and control strategies, establish emergency price monitoring team, establish surplus material monitoring mechanism and network public opinion monitoring. It mainly includes price monitoring, supply monitoring, and network public opinion monitoring	18
	Timely response	In response to panic buying events that have occurred, timely respond and publish authoritative information, mainly in the form of a speech by the Department Director to promote price stability and sufficient reserves	53
Society	Positive guidance	Social forces such as news media and online celebrities spread positive information, so as to guide people not to participate in panic buying. For example, release sufficient information on materials after field investigation, and release experts' Analysis on material supply, etc	29
	Negative reinforcement	Social forces such as news media and online celebrities refute rumors about negative information, so as to guide people not to participate in panic buying. For example, labeling rumor information, increasing the exposure of rumor refutation information, etc	8
Individual	Psychological counseling	Psychologists release ways to reduce stress on online social media, calling on people to not panic too much and maintain a good psychological state to alleviate people's panic psychology	66

It can be seen from Supplementary Figure 1 that February, April 2020 and January 2021 accumulated the most relevant news, which is consistent with the actual panic buying event. In addition, government intervention measures are diverse in different periods. In January 2020 when COVID-19 initially appeared, the government mainly adopted timely response, and authoritative information was transparent, which gave the public “reassurance.” In February 2020, psychological counseling had the most relevant news. This is because after the accumulation of emotions in January, the level of panic and anxiety among the people has risen to an unprecedented level. The government needed to release psychological counseling from many experts and scholars, advocating the people to cope with it calmly without being overly anxious. In April 2020, in order to alleviate people's panic about the increase in food prices, the government took timely measures, responding to the country's grain storage situation immediately, clarifying that the food supply was sufficient and the price would not rise, and supplemented by psychological counseling. In August 2020, although there were no large-scale panic buying, due to the impact of floods and the fluctuation of international grain prices, the operation of grain market has experienced phased fluctuations, which has attracted widespread attention from the society. The government has responded timely. There was no panic buying incident in the follow-up. In January 2021, due to the escalation of the Shijiazhuang epidemic situation, panic buying reappeared, the government has implemented multiple measures, released authoritative information, and combined with offline real scenes to positively guide the public, conducted psychological counseling so as to prevent the spread of rumors. In addition, at this stage, the government's prevention and control measures have also been strengthened, and mechanisms such as price monitoring and online public opinion monitoring have been established. Based on the above analysis, it can be found that: (1) social intervention measures are diversified, which can be divided into five categories: government prevention and control, timely response, positive guidance, negative reinforcement, and psychological counseling; (2) different types of intervention measures are required for different situations.

Based on the above content and the three intervention directions of government intervention, interpersonal intervention and business behavior intervention pointed out by some scholars in the literature review, combined with the causes of panic buying, the final design of panic buying social intervention measures is shown in Table 3.

**Table 3:** Panic buying social intervention measures.

**Category**	**Factor**	**Measure**	**Meaning**
External environment	Panic buying atmosphere	Information guidance	From the positive aspects of field visits, expert analysis, and correct understanding of rumors, or from the negative aspects of punishing rumors, punishing price-raising behaviors, and other negative aspects to guide the public not to participate in panic buying
		Information review	Netizens (ordinary netizens, opinion leaders) who participate in offline purchases may publish their experience. If it does not match the actual supply of materials, the information spread will be stopped; otherwise the information spread will be encouraged
	Official material measure	Official response	Timely respond and publish authoritative information of panic buying incidents that have already occurred
		Supply monitoring	Monitor the supply of materials in the market and regulate timely to prevent problems such as shortage of materialS
Internal environment	Panic emotion	Psychological counseling	Psychologists give suggestions to eliminate panic and get rid of panic psychology

## Model Construction

Monte Carlo's simulation (46) is used for modeling. Monte Carlo simulation, also known as statistical experiment method, is a calculation method based on probability theory and statistical theory. The complex real problem is transformed into a probability model, and the statistical simulation is realized by computer to obtain the approximate solution of the problem. It is widely used in financial engineering, macroeconomics and other fields. This method has clear and concise structure and strong flexibility. It uses programming to simulate individual motion, which can directly track the behavior of each individual at each time, and can more truly simulate the motion process of individuals through random sampling method. Meanwhile, Agent is used to represent individual nodes in the network, and the network scale is set to *N*, that is, there are *N* netizen nodes in the network. The research framework is shown in Supplementary Figure 2.

This article selects the BA network (47) as the node interaction network. BA scale-free network is a power-law distribution network model, which can simulate real social networks. In the generation process of BA network, the network scale continues to expand, and new nodes tend to connect with nodes with high connectivity, which is consistent with the law that people are more inclined to communicate with more influential people in life. Analyzes the formation of individual panic buying behavior in the model at first, then constructs a social intervention mechanism, and corresponds each measure in the intervention mechanism to the formation process of individual panic buying behavior, so that it can play intervention role in panic buying behavior.

Based on the social intervention measures in Table 3, this article constructs a social intervention model for panic buying behavior in an emergency situation. The idea of model construction is shown in Supplementary Figure 2.

From the perspective of panic buying behavior, on the one hand, the stimulation of external information has increased people's demand for materials and safety. On the other hand, under the dual pressure of neighboring panic buyers and the individual's own demands, the panic emotion is also increasing. Therefore, no matter from the perspective of rational demand or perceptual emotional perspective, people tend to buy materials. Under normal circumstances, after people make large-scale panic buying in supermarkets, if the supplies in the supermarket continue to decrease and the replenishment is not timely, shelves may be empty. The batch of people may post their acquired information of the reality on the Internet, affecting more people with the panic, thus forming a vicious circle.

From the perspective of social intervention: (1) the materials supply should be monitored offline to timely replenish goods, ensure sufficient offline materials, thus making buyers relieved. (2) The comments on the Internet about panic buying should be reviewed to check whether the opinions conveyed are consistent with the real supply situation, and marked as rumors or truth. (3) Information guidance is necessary to encourage netizens not to believe in rumors but believe in the truth. (4) Officials should timely understand the reasons for the panic buying and respond to the concerns of the people. (5) Psychologists and other social forces provide psychological counseling to netizens to alleviate their panic. The parameters and variables involved in the model are shown in Tables 4, 5.

**Table 4:** Parameter description.

**Name**	**Description**
*N*	Total number of persons
α	Weight of material needs (physiological needs) in individual needs
β	Weight of safety needs in individual needs
μ	Influence parameter that people see and hear about material situation
*M*_0_	Initial value of material supply
*S*_0_	Initial value of safety supply
*Con*(*i*)	Conformity of individual *i*
*d*_*SD*_	Supply and demand threshold
*d*_*A*_	Panic buying threshold
θ_1_	Influence weight of individual demand on panic buying behavior
θ_2_	Influence weight of panic emotion on panic buying behavior
γ	Influence parameters of information authenticity
λ_1_	Influence parameters of official response on material demand
λ_2_	Influence parameters of psychological counseling on material demand
*Q*_*move*_(*t*)	Amount of materials moved from other places at time *t*
*Gov*(*t*)	Intensity of the government's official response to material supply at time *t*
*Str*_*PR*_(*t*)	Adjustment of panic emotion by psychological counseling measures at time *t*
*t*_*exam*_	Delays caused by information review
*t*_*timelyG*_	Timeliness of official response
*t*_*timelyP*_	Timeliness of psychological counseling
*TR*(*j*)	Trust in neighbor *j*
*TR*_*gov*_	Trust in government
*TR*_*ol*_	Trust in opinion leader

**Table 5:** Parameter description.

**Name**	**Description**
*A*_*i*_(*t*)	Attitude of individual *i* toward panic buying behavior at time *t*
*M*_*i*_(*t*)	Material demand of individual *i* at time *t*
*S*_*i*_(*t*)	Safety demand of individual *i* at time *t*
*E*_*i*_(*t*)	Panic emotion of individual *i* at time *t*
*Self*_*i*_(*t*)	Self experience of individual *i* at time *t*
*State*_*i*_(*t*)	Buying state of individual *i* at time *t*
*order*_*i*_(*t*)	Panic buying order of individual *i* at time *t*
*I*_*i*_*net*_(*t*)	Influence of the information released by the neighbor of individual *i* at time *t* on its material demand
*N*_*i*_(*t*)	Neighboring number of individual *i* at time *t*
*NI*_*i*_(*t*)	Panic buying neighboring number of individual *i* at time *t*
*NI*(*t*)	Total number of panic buyers at time *t*
*F*_*i*_(*t*)	Influence of neighbors of individual *i* at time *t*
*Q*(*t*)	Total amount of social materials at time *t*
*Q*_*i*_(*t*)	Amount of social materials that individual *i* sees at time *t*
*I*_*type*_	Information review results
*TF*_*j*_(*t*)	Authenticity of the information sent by sender *j* at time *t*
*I*_*gov*_(*t*)	Influence of the official response at time *t* on individual material needs
*PR*(*t*)	Influence of psychological counseling measures at time *t* on individual panic

### Panic Buying

Panic buying is a behavioral decision. At the initial moment, an individual's panic buying behavior is affected by his needs and panic. Online news and surrounding atmosphere will affect individual's needs and panic. External news may report the insecurity of the external environment and the increasing lack of social materials, which makes people's demands for safety and physiological materials gradually increase. Moreover, people's panic buying in supermarket will also affect the people around them. Under the combined influence of the increasingly deepening panic buying atmosphere and their own demand pressure, the people's panic will also increase. The interaction of individual needs and panic makes individuals change from “no panic buying” state to “panic buying” state.

Based on this, *A*_*i*_(*t*) represents the attitude of individual *i* to panic buying behavior at time *t, A*_*i*_(*t*) belongs to [0, 1], the higher the value is, the higher the individual's support is for the buying behavior, the calculation formula is as follows:


(1) $$\begin{array}{l} A_{i}\;(t)=\theta_{1}*\left(a*M_{i}\;(t)+\beta *\left(1-S_{i}\;(t)\right)\right)+\theta_{2}*E_{i}\;(t) \end{array}$$

where *M*_*i*_(*t*) represents material demand of individual *i* at time *t, S*_*i*_(*t*) represents safety demand of individual *i* at time *t* and *E*_*i*_(*t*) represents panic emotion of individual *i* at time *t*. θ_1_ and θ_2_ are influence weight of individual needs and panic emotion on panic buying behavior, and θ_1_ + θ_2_ = 1. α and β represent the weight of material needs and safety needs in individual needs, and α + β = 1. According to Maslow's hierarchy of needs theory, physiological needs are higher than safety needs, so α > β.

Generally speaking, the higher the value of panic emotion is, the more irrational the individual is, and the stronger the effect of emotion on the individual's panic buying behavior is. Correspondingly, individual's demand has a weaker effect on buying behavior. Therefore, the value of panic emotion can be used to measure θ_1_ and θ_2_, and the formula is as follows:


(2) $$\begin{array}{l} \{\begin{array}{c} \theta 1=|1-E_{i}\;(t)|\\ \theta 2=|E_{i}\;(t)| \end{array} \end{array}$$

When the panic buying attitude *A*_*i*_(*t*) exceeds the panic buying threshold, the individual panic buying state changes from “no panic buying” to “panic buying.” *State*_*i*_(*t*) is used to mark the panic buying state of individual *i* at time *t*, and its value is 0 or 1. *State*_*i*_(*t*) = 0 means that the individual is not a panic buyer, *State*_*i*_(*t*) = 1 means that the individual is currently panic buyer. The calculation formula is as follows:


(3) $$\begin{array}{l} State_{i}\;(t)=\{\begin{array}{c} 0,\text{ if }A_{i}\;(t)<d_{A}\\ 1,\text{ if }A_{i}\;(t)\geq d_{A} \end{array} \end{array}$$

where *d*_*A*_ is the panic buying threshold.

#### Individual Needs

People's Needs for materials and safety will be affected by outside information. During the epidemic, this information was mainly spread through online channels. When the information about materials and safety issues is received by people, everyone will synthesize the information they receive to form their own judgments on whether the external materials are sufficient and whether the external environment is safe. Here, the material demand *M*_*i*_(*t*) and the safety demand *S*_*i*_(*t*) are introduced.

##### Material Need *M*_*i*_(*t*)

*M*_*i*_(*t*) represents material need of individual *i* at time *t, M*_*i*_(*t*)∈(0, 1). The larger the value is, the higher the need for materials is, and people are more prone to panic buying. At the initial moment, the initial value of the individual material need *M*_0_ is set according to the external news. For example, when the material is in short supply in a news report, the *M*_0_ is larger, and when the material is sufficient in the news report, *M*_0_ is smaller. At this time, some individuals will form their own knowledge about the supply of materials based on their experience. This kind of personal experience will not only change their own judgments about material needs in the next moment, but also indirectly affect others through the information they release. Therefore, its calculation formula is as follows:


(4) $$\begin{array}{l} M_{i}\;(t)=\{\begin{array}{c} M_{0},\text{ if }t=1\\ M_{i}\left(t-1\right)+Self_{i}\;(t),\text{ if }t>1\cup State_{i}\left(t-1\right)=1\\ M_{i}\left(t-1\right)+I_{i\text{\_}net}\;(t),\text{ if }t>1\cup State_{i}\left(t-1\right)=0 \end{array} \end{array}$$

where *M*_0_ is the initial value of material needs. If an individual participates in the panic buying at the last time, it will form a judgment on the material demand through the situation it sees offline, which is *Self*_*i*_(*t*−1). *Self*_*i*_(*t*−1) represents self experience of individual *i* at time *t*-1. If an individual does not participate in the panic buying at the last time, the judgment of material demand is formed through the information released by the surrounding neighbors, which is *I*_*i_net*_(*t*). *I*_*i_net*_(*t*) represents influence of the information released by the neighbor of individual *i* at time *t* on its material needs.

##### Self Influence *Self*_*i*_(*t*)

At the initial moment, the amount of social materials is 1 unit, which represents the amount that can satisfy all people's purchase of basic living materials once. With the occurrence of panic buying, the amount of social materials will continue to decrease. When it is lower than the supply and need threshold, it means that the actual supply of materials is insufficient. At this time, the people's need for materials will rise, and vice versa. Here, *Self*_*i*_(*t*) represents self experience of individual *i* at time *t*, and its calculation formula is as follows:


(5) $$\begin{array}{l} Self_{i}\;(t)=\{\begin{array}{c} 0,\text{ if }State_{i}\left(t-1\right)=0\\ \mu \left(d_{SD}-Q_{i}\;(t)\right),\text{ if }State_{i}\left(t-1\right)=1 \end{array} \end{array}$$

where μ is an influence parameter that people see and hear about material situation, *State*_*i*_(*t*−1) represents buying state of individual *i* at time *t*-1, *d*_*SD*_ represents supply and need threshold, *Q*_*i*_(*t*) represents amount of social materials that individual *i* perceives at time *t*. If the individual is in a panic buying state, when *Q*_*i*_(*t*) < *d*_*SD*_, the material supply is insufficient and *Self*_*i*_(*t*) > 0, people's material needs will increase. When *Q*_*i*_(*t*) > *d*_*SD*_, the material supply is sufficient and *Self*_*i*_(*t*) < 0, people's material needs will decrease. When *Q*_*i*_(*t*) = *d*_*SD*_, one's own experience has no effect on the people's material needs.

##### Influence of Neighbor' Information *I*_*i*_*net*_(*t*)

Panic buyers will spread their experience about the materials to other individuals in the form of information release, so the individuals not participating in the panic buying will be affected by the information of these neighbor nodes when judging whether the materials are sufficient. *I*_*i_net*_(*t*) represents influence of the information released by the neighbor of individual *i* at time *t* on its material needs, and its calculation formula is as follows:


(6) $$\begin{array}{l} I_{i\text{\_}net}\;(t)=\frac{\sum_{j=1}^{j=NI_{i}}\left(A_{j}\;(t)-d_{A}\right)\;*\;TR\;(j)}{NI_{i}\;(t)} \end{array}$$

where *A*_*j*_(*t*) represents attitude of individual *i* toward panic buying behavior at time *t, d*_*A*_ is panic buying threshold, and *TR*(*j*) is trust in neighbor *j*. Notice that netizens are divided into two types: ordinary netizens and opinion leaders, and the people have different levels of trust in these two types of subjects. *NI*_*i*_(*t*) represents panic buying neighboring number of individual *i* at time *t*. *M*_*i*_(*t*-1) represents the panic buying need of individual *i* at *t*-1. If the average attitude value of the neighbors who are panic buyers is smaller than the individual's panic buying attitude, then *I*_*i*_*net*_(*t*) < 0, and the individual's material need will be weakened.

##### Safety Need *S*_*i*_(*t*)

In addition to material need, individual need also include security need. *S*_*i*_(*t*) represents the safety need of individual *i* at time *t, S*_*i*_(*t*)∈(0, 1). The larger the value is, the higher the individual's vigilance to the external environment will be, the more insecure the external environment will be, and the less the people are willing to go out. Since most safety information is disclosed in official news, its calculation formula is as follows:


(7) $$\begin{array}{l} S_{i}\;(t)=S_{0} \end{array}$$

where *S*_0_ is the initial value of material need.

#### Panic Emotion

The formation of panic is affected by the needs of the individual from the internal cause and the surrounding individuals from the external cause. All kinds of epidemic-related information on the Internet can stimulate the actual needs of the people. When these actual needs are not met, individuals will feel panic. In addition, when most neighboring people begin to make panic buying, the surrounding atmosphere further promotes the individual's panic. Based on the above analysis, *E*_*i*_(*t*) represents the panic value of individual *i* at time *t, E*_*i*_(*t*)∈(0,1), the higher the value is, the higher the panic degree will be, and the calculation formula is as follows:


(8) $$\begin{array}{l} E_{i}\;(t)=\alpha *M_{i}\;(t)+\beta *S_{i}\;(t)+F_{i}\;(t) \end{array}$$

where α and β are the weights of material needs (physiological needs) and safety needs in individual needs, α + β = 1. Since the material needs are higher than the safety needs in Maslow's hierarchy of needs, α > β; *M*_*i*_(*t*) and *S*_*i*_(*t*) are the material needs and safety needs of individual *i* at time *t*, respectively; *F*_*i*_(*t*) means influence of neighbors of individual *i* at time *t*.

The influence of neighboring individuals is related to the number of surrounding individuals who take panic buying and individual conformity. The calculation formula is as follows:


(9) $$\begin{array}{l} F_{i}\;(t)=\frac{NI_{i}\;(t-1)}{N_{i}\;(t-1)}*Con\;(i) \end{array}$$

where *N*_*i*_(*t*−1) the number of neighbor of individual *i* at *t*-1, *NI*_*i*_(*t*−1) represents panic buying neighboring number of individual *i* at time *t*−1. Generally speaking, more neighbors around an individual who take panic buying represents it is easier to cause panic. *Con*(*i*) represents the conformity degree of individual *i*, which is related to the individual's growth environment, educational background and other social factors.

### Amount of Social Materials

The amount of social materials represents the total amount of materials in a certain area, and the amount of social materials will decrease when the number of panic buyers increases.

On the whole, *Q*(*t*) represents the total amount of social materials at time *t*, and the total amount of social materials at the initial moment is counted as 1, that is, *Q*(*t*) = 1. The calculation formula is as follows:


(10) $$\begin{array}{l} Q\;(t)=\{\begin{array}{c} 1,\;t=1\\ Q\left(t-1\right)-\frac{1}{N}*NI\;(t-1),t\geq 2 \end{array} \end{array}$$

where *Q*(*t*−1) represents the total amount of social materials at *t*−1, *N* represents the total number of people, and *NI*(*t*−1) represents the total number of panic buyers at *t*−1.

For individuals, the amount of social materials seen at the same time may vary due to the different order of panic buying. For example, the first panic buyer at the same time sees more social materials than the last, so *Q*_*i*_(*t*) represents the amount of social materials that individual *i* sees at time *t*, and its calculation formula is as follows:


(11) $$\begin{array}{l} Q_{i}\;(t)=Q\left(t-1\right)-\frac{1}{N}*order_{i}\;(t),\text{ if }Stete\;(i,t-1)=1 \end{array}$$

where *order*_*i*_(*t*) represents panic buying order of individual *i* at time *t*.

### Social Intervention Mechanism

#### Offline Intervention Mechanism

The offline intervention mechanism is mainly to monitor the supply situation. Measure whether the supply of materials is sufficient by monitoring panic buying and the amount of social materials. When the supply of materials is out of balance, the materials will be regulated in time to ensure the supply of offline materials, which will have an impact on the people's own knowledge. On the one hand, this can directly alleviate the concerns of panic buyers. On the other hand, it can also pass on the sufficient information of the materials released by these people to other people, forming a positive impact. Therefore, under supply monitoring, the total amount of social materials *Q*(*t*) can be supplemented on the basis of formula (10) as follows:


(12) $$\begin{array}{l} Q\;(t)=\{\begin{array}{c} Q\;(t)+Q_{move}\;(t),\text{ if }Q\;(t)<d_{SD}\\ Q\;(t),\text{ if }Q\;(t)\geq d_{SD} \end{array} \end{array}$$

where *Q*_*move*_(*t*) represents amount of materials moved from other places at time *t*.

#### Online Intervention Mechanism

Online intervention mechanisms include information review, information guidance, official response and psychological counseling.

Information review refers to the authenticity review of material information published by netizens. Some netizens will choose to post their information online after panic buying. If the information posted by the netizen does not match the actual supply of materials, the review will be rejected, and such information will be marked as a rumor. Otherwise, the review will be passed. Audit can improve the authenticity of the information. However, since the audit takes time to process, it will slow down the speed of information dissemination. *I*_*type*_ represents the audit result, *I*_*type*_ = 1 represents a rumor, and *I*_*type*_ = 2 represents true information. The calculation formula is as follows:


(13) $$\begin{array}{l} Itype_{i}\;(t)=\{\begin{array}{c} 1,\text{ if }\left(A_{i}\;(t)-d_{A}\right)*\left(d_{SD}-Q\;(t)\right)<0\;\\ 2,\text{ if }\left(A_{i}\;(t)-d_{A}\right)*\left(d_{SD}-Q\;(t)\right)\geq 0 \end{array} \end{array}$$

where *A*_*i*_(*t*) represents the panic buying attitude of individual *i* at time *t, d*_*A*_ is the panic buying threshold, *Q*(*t*) is the total amount of social materials at time *t*, and *d*_*SD*_ is the supply-demand threshold. The information released by publisher *i* is presented with its *A*_*i*_(*t*). When *A*_*i*_(*t*) < *d*_*A*_ and *d*_*SD*_ < *Q*(*t*), it means that *i*'s attitude tends no panic buying and the material supply is sufficient, and the information is authentic, at this time, (*A*_*i*_(*t*)-*d*_*A*_) * (*d*_*SD*_-*Q*(*t*))>0, *I*_*type*_ = 2; similarly, when the individual's buying tendency does not match the real situation, the information is false. At this time (*A*_*i*_(*t*)-*d*_*A*_) * (*d*_*SD*_-*Q*(*t*)) < 0, *I*_*type*_ = 1.

Information guidance includes positive and negative information guidance, and its premise is information review. When the information is marked as a rumor, netizens are guided to stop the rumor, so that the netizens reject the information; when the information is marked as true, the netizens are guided to correctly recognize the information, so that the netizens trust the message. Therefore, formula (6) can be rewritten as:


(14) $$\begin{array}{l} I_{i\text{\_}net}\;(t+t_{exam})=\frac{\sum_{j=1}^{j=NI_{i}}\left(A_{j}\;(t)-d_{A}\right)\;*\;TR\;(j)\;*\;TF_{j}\;(t)}{NI_{i}\;(t)} \end{array}$$

where *t*_*exam*_ represents the delay time caused by information review, *TF*_*j*_(*t*) is the authenticity influence of the information sent by sender *j* at time *t*, and its calculation formula is as follows:


(15) $$\begin{array}{l} TF_{j}\;(t)=\{\begin{array}{c} -\gamma ,\text{ if }Itype_{j}\;(t)=1\\ \gamma ,\text{ if }Itype_{j}\;(t)=2 \end{array} \end{array}$$

where γ represents the influence parameter of the information authenticity.

The official response is to respond to and publish authoritative information about the panic buying phenomenon that has already occurred. The main form is mostly “department head makes speech, clarifying the price stability and sufficient reserves,” so that the public can form a judgment on the materials demand. In addition to non-governmental information channels, there are also official government information channels, making netizens' judgments more comprehensive. Therefore, formula (4) can be rewritten as:


(16) $$\begin{array}{l} M_{i}\;(t)=\{\begin{array}{c} M_{0},\text{ if }t=1\\ M_{i}\;(t-1)+Self_{i}\;(t)-\lambda_{1}*I_{gov}\;(t),\\ \text{if }t>1\cup State_{i}\;(t-1)=1\\ M_{i}\;(t-1)+I_{i\text{\_}net}\;(t)-\lambda_{1}*I_{gov}\;(t),\\ \text{if }t>1\cup State_{i}\;(t-1)=0 \end{array} \end{array}$$

where *I*_*gov*_(*t*) represents the influence of the official response at time t on individual material demand, λ_1_ represents influence parameter. The calculation of *I*_*gov*_(*t*) is as follows


(17) $$\begin{array}{l} I_{gov}\;(t+t_{timelyG})=Gov\;(t)*TR_{gov} \end{array}$$

where *t*_*timelyG*_ represents timeliness of official response. The larger value of *t*_*timelyG*_ indicates that the response is less timely. *Gov*(*t*) indicates the degree of the official response to the material supply at time *t* and *Gov*(*t*)∈(0, 1). The larger value represents the strong degree of response, which can better relieve public's concern. *TR*_*gov*_ indicates public's trust to officials.

Psychological releasing measure is an adjustment mechanism for panic emotion, which is mainly manifested in the psychological counseling given by social forces (opinion leaders). For example, psychologists post tweets to guide netizens in panic, point out ways to eliminate panic emotions, and advocate people to maintain a peaceful state of mind, etc. This intervention mechanism can help people from the emotional perspective. Therefore, formula (8) can be rewritten as:


(18) $$\begin{array}{l} E_{i}\;(t)=\alpha *M_{i}\;(t)+\beta *S_{i}\;(t)+F_{i}\;(t)-\lambda_{2}\left(t\right) \end{array}$$

where *PR*(*t*) represents the influence of psychological releasing measures on individual panic at time *t*, and λ_2_ is its influence parameter. The specific calculation formula of *PR*(*t*) is as follows:


(19) $$\begin{array}{l} PR\;(t+t_{timelyP})=Str_{PR}\;(t)*TR_{ol} \end{array}$$

where *t*_*timelyP*_ represents the timeliness of psychological counseling measures. The larger the value is, the less timely the response is. *Str*_*PR*_(*t*) represents the adjustment strength of psychological counseling measures to panic at time *t, Str*_*PR*_(*t*)∈(0, 1). The larger the value is, the greater the adjustment degree is, and the more it can alleviate the people's panic. *TR*_*ol*_ represents the people's trust in opinion leaders.

**Figure 1:**
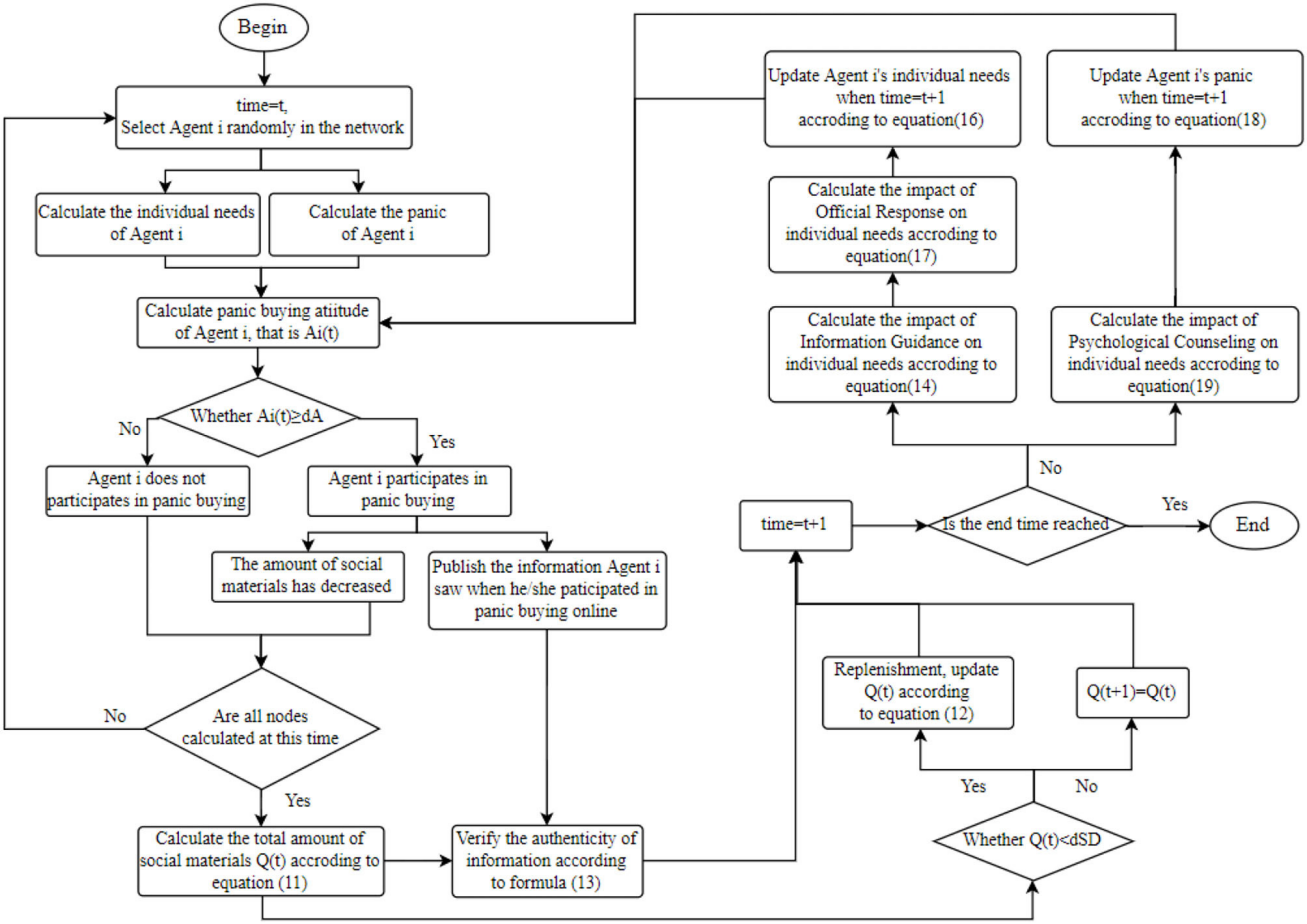
Evolution flow chart.

Based on the above analysis, the evolution process of the social intervention model for panic buying behavior is shown in Figure 1.

## Simulation Experiment

This section uses MATLAB to simulate the model constructed above to analyze the effects of offline interventions (supply monitoring) and online interventions (information review, information guidance, official response, psychological counseling) on panic buying.

The initial network of the simulation experiment is a BA scale-free network with a node size of 1,000. The nodes are divided into two types: ordinary nodes and opinion leader nodes. The top 5% of the connected nodes are set as opinion leader nodes, and the other nodes are ordinary nodes. According to the central limit theorem, a person's height, shoe size, and environment all obey a normal distribution. Therefore, the individual conformity degree *Con*(*i*) is set to obey the normal distribution of *N* ~ (0.5, 0.15), and the randomly generated number >1 is set to 1, and the number <0 is set to 0, so that the parameter is mapped to the [0, 1] interval. The mean value of 0.5 indicates that the conformity of most individuals in the group is in the middle, and the variance of 0.15 is to make all the numbers in the range of [0, 1] get the probability value. It is assumed that the initial value *M*_0_ obeys the normal distribution of *N* ~ (0.5, 0.15), and the initial value *S*_0_ obeys the normal distribution of *N* ~ (0.5, 0.15). According to Maslow's hierarchy of needs theory, material needs are more basic and important than safety needs. Therefore, α is 0.6, β is 0.4. The other parameters are set as follows: μ is 0.2, *d*_*SD*_ is 0.65, and *d*_*A*_ is 0.6.

**Figure 2:**
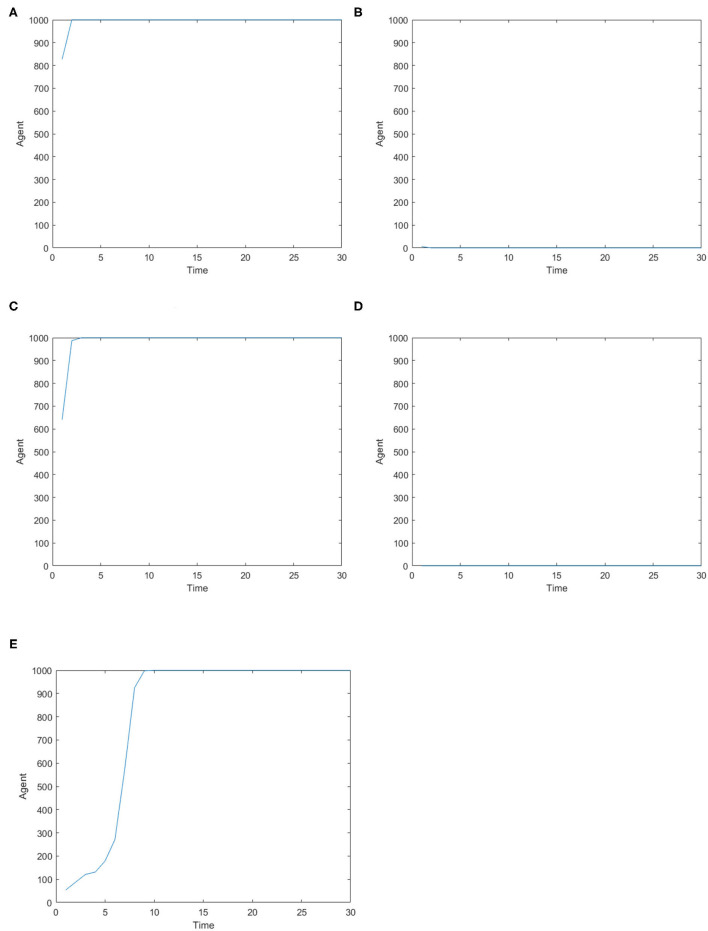
The number of panic buyers under different need situations without intervention changes over time. **(A)** High safety need, high material need. **(B)** High safety need, low material need. **(C)** Low safety need, high material need. **(D)** Low safety need, low material need. **(E)** Medium safety need, medium material need.

Figure 2 is the number of panic buyers under different needs situations without intervention changes over time. According to formula (3), when the value of *A*_*i*_(*t*) exceeds *d*_*A*_, the individual will take panic buying and *State*_*i*_(*t*) is 1, otherwise *State*_*i*_(*t*) is 0. By calculating the number of individuals with *State*_*i*_(*t*) = 1 at each moment, the number of panic buyers at different moments was counted. Set the low needs to follow the normal distribution of *N* ~ (0.2, 0.15), the medium needs to follow the normal distribution of *N* ~ (0.5, 0.15), and the high needs to follow the normal distribution of *N* ~ (0.8, 0.15). Figures 2A–E respectively represent (high safety needs, high material needs), (high safety needs, low material needs), (low safety needs, high material needs), (low safety needs, low material needs), (medium safety needs, medium material needs) situations. It can be seen from Figures 2B,D that when the material needs is low, there is no panic buying; from Figures 2A,C,E, it can be seen that when the material needs is medium or high, panic buying is triggered in varying degrees. Therefore, the follow-up discussion will focus on the three situations of high safety needs and high material needs, low safety needs and high material needs, and medium safety needs and material needs.

### The Impact of Supply Monitoring on Panic Buying

Supply monitoring refers to monitoring the offline supply of materials. When the supply is found to be insufficient, it is timely dispatched from other places, so as to ensure that people can always see the supply of materials offline, and there will be no pictures of empty supermarket shelves online, alleviating the urge of panic buying. In order to analyze the impact of supply monitoring measures on panic buying, different initial material requirements are set, and 1 unit of material is automatically replenished every time the supply and demand threshold is lowered, that is, *Q*_*move*_(*t*) is 1.

**Figure 3:**
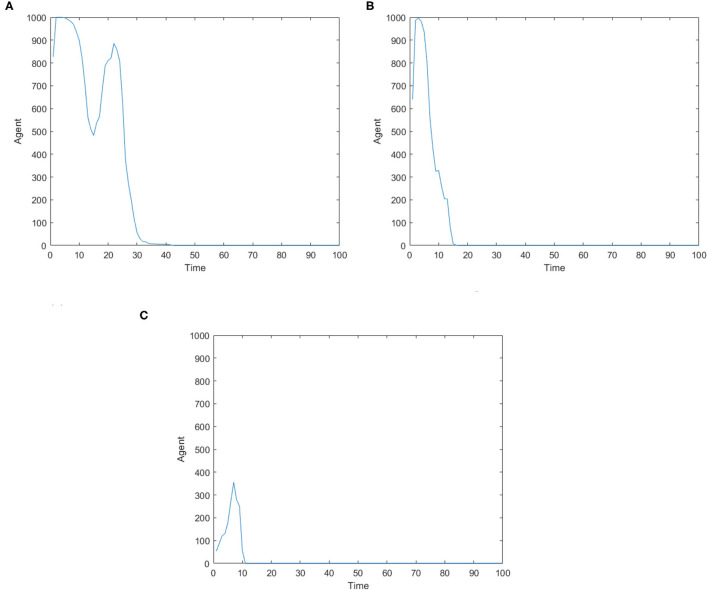
The number of panic buyers over time under different need conditions under the monitoring intervention of supply situation. **(A)** High safety need, high material need. **(B)** Low safety need, high material need. **(C)** Medium safety need, medium material need.

**Figure 4:**
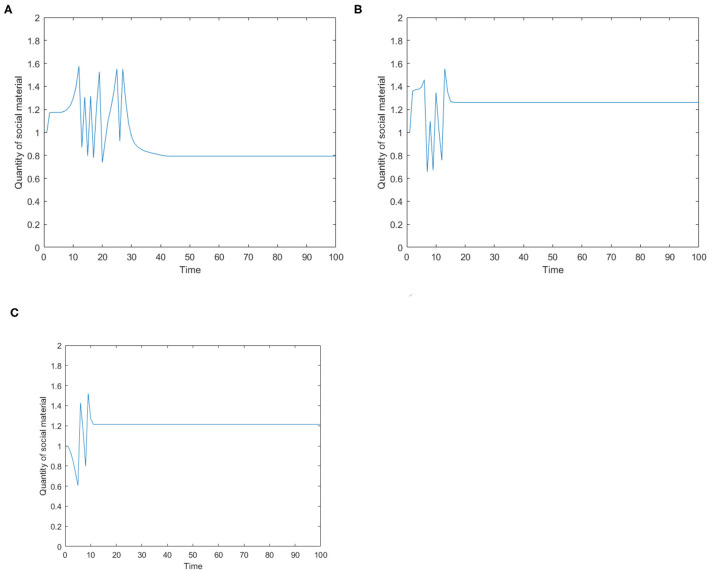
The amount of social materials over time under different need conditions under the monitoring and intervention of the supply situation. **(A)** High safety need, high material need. **(B)** Low safety need, high material need. **(C)** Medium safety need, medium material need.

**Figure 5:**
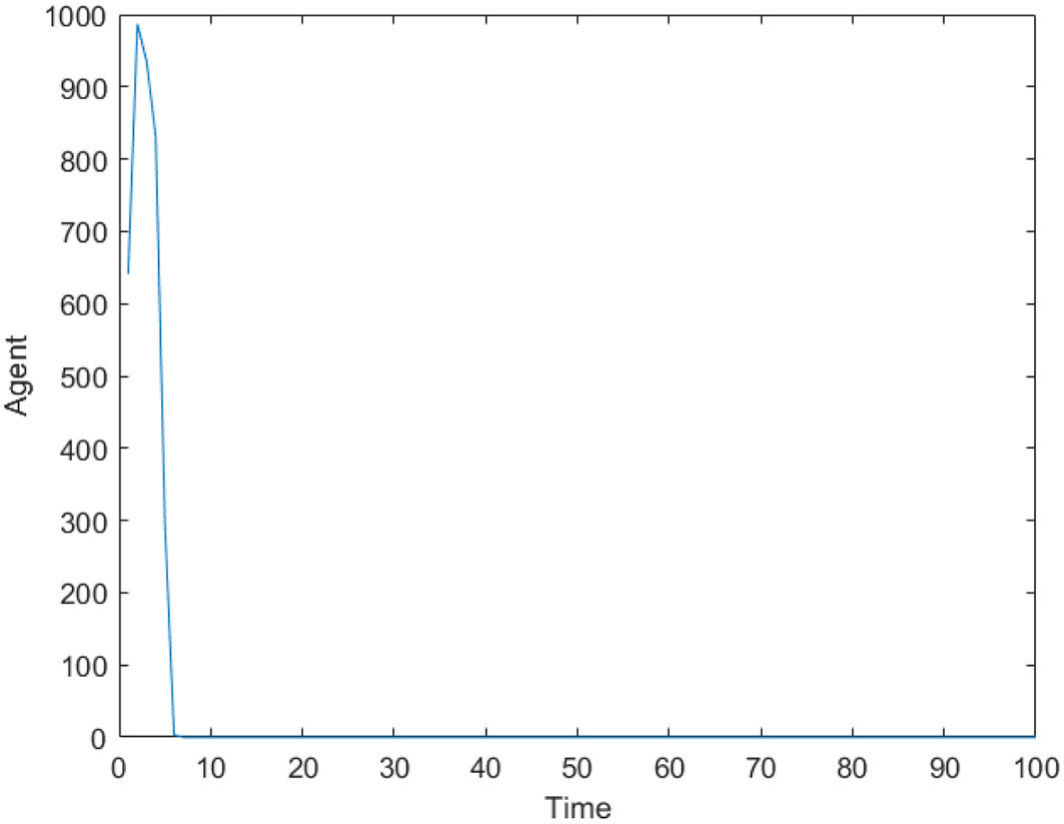
The changes of number of panic buyers over time after adjusting *Q*_*move*_(*t*) = 2 based on the situation in Figure 3A.

Figure 3 is the number of panic buyers over time under different needs conditions under the monitoring intervention of supply situation. Comparing Figures 2A,C,E, 3A–C respectively, we find that supply monitoring can effectively curb panic buying behavior, and the lower needs brings better effect. Figure 4 is the amount of social materials over time under different needs conditions with the monitoring and intervention of the supply situation. It can be seen from the figure that in the three cases, material replenishment was mobilized 6, 4, and 2 times, indicating that the lower needs represents the less amount of materials that needs to be mobilized. It can be seen from Figure 4 that when the initial need is high, the number of panic buyers gradually decreased with the monitoring measures of the supply situation at first, but then another fluctuation occurred at time = 15. This may be related to the volume of a single transfer. Since the simulation sets the single supply transfer volume to be fixed at 1, even if 1 unit of materials is transferred at time = 15, the volume of materials that the people need to snap up may still not be met, which caused another wave of panic buying. To verify this phenomenon, *Q*_*move*_(*t*) in Figure 3A is set from 1 to 2, and the simulation result is shown in Figure 5.

By comparing Figure 5 with Figure 3A, it can be found that the phenomenon of panic buying disappeared quickly after the increase of the amount of material mobilization, which indicates that the quantity of supply transfer has impact on the alleviation of panic buying. Therefore, when the relevant departments monitor the supply situation, relevant departments also need to pay attention to the needs changes of the quantity of materials at different time moments, and dynamically adjust the quantity of materials mobilized, so as to alleviate the panic buying phenomenon more quickly.

### Impact of Information Review and Guidance Mechanism on Panic Buying

Soon after the panic buying incident, a large amount of related information was posted on social networks, and people paid special attention to the information on empty supermarket shelves, but the information was mixed and unverified. Apparently, and the dissemination of false information was very likely to inspire panic among the people. If there is a negative impact, the use of information review and guidance mechanisms can effectively alleviate the occurrence of this situation. Information review is the prerequisite for information guidance. It is necessary that the authenticity of the information released by the public is judged, the authenticity of the information is identified and verified, the publicity of the real information is increased, and the false information is criticized to encourage the public to be aware of the truth.

**Figure 6:**
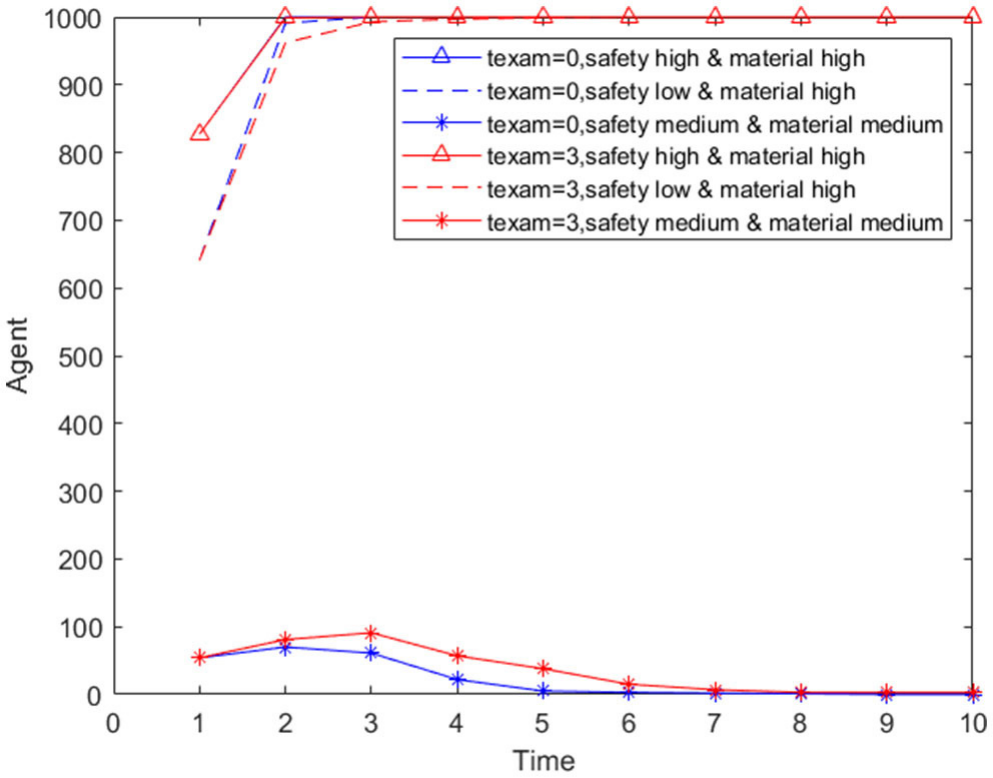
The number of panic buyers over time under different need based on information review and guidance.

People's needs situation when panic buying occurs will have an impact on the intervention effect of information review and guidance. The delay time *t*_*exam*_ caused by information review may also affect the intervention effect. Therefore, the effect of different needs conditions and different review delay times on the number of panic buyers is simulated. Setting γ is 1, and the result is shown in Figure 6.

Comparing Figures 2A,C,E, 6, it can be found that the intervention effect of the information review and guidance mechanism on panic buying varies with the actual situation. The intervention effect is better when the safety and material needs are in the medium, while the effect is worse when material needs is higher. At the same time, the longer *t*_*exam*_ caused by the information review indicates longer the panic buying duration.

**Figure 7:**
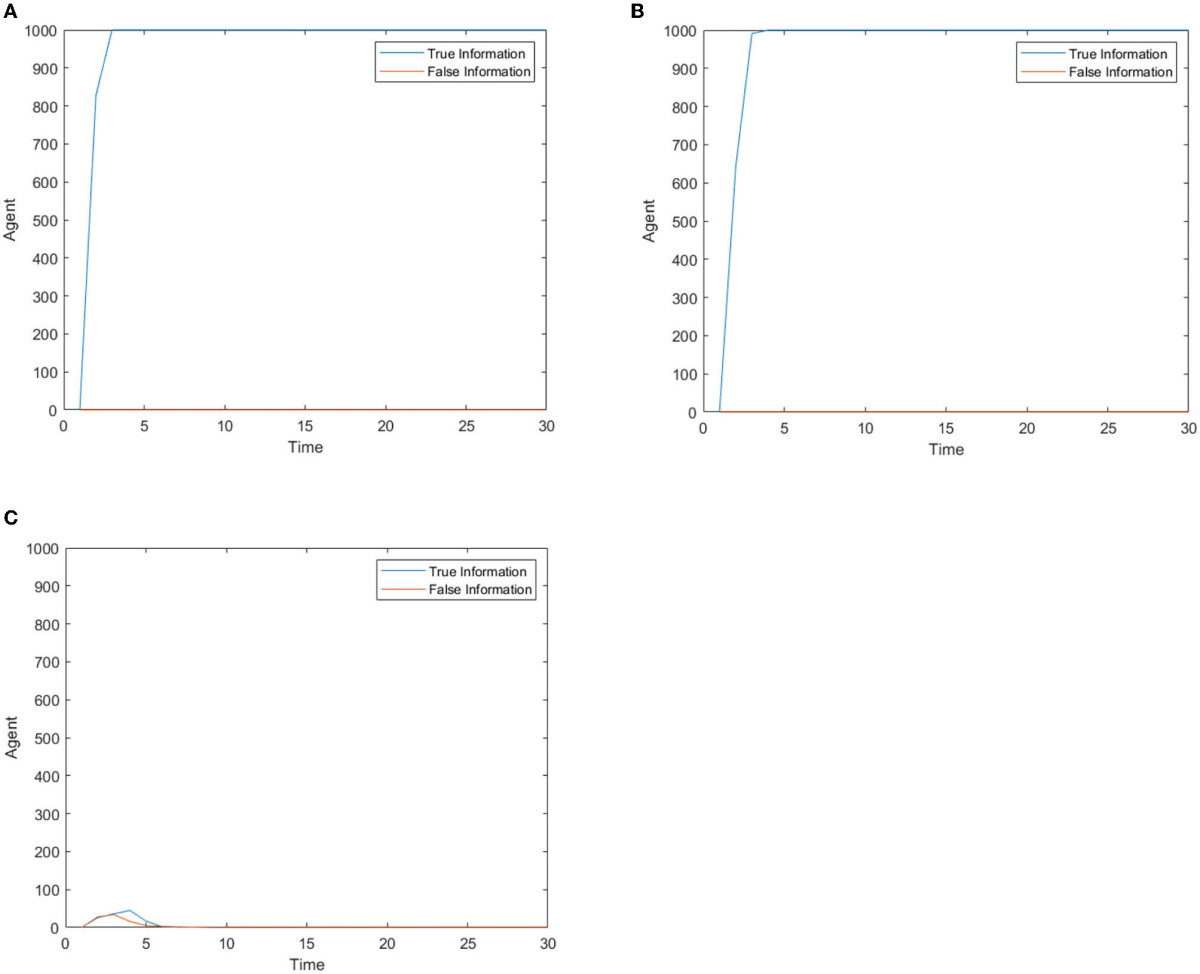
The number of people posting true or false information over time. **(A)** High safety need, high material need. **(B)** low safety need, high material need. **(C)** Medium safety need, medium material need.

Figure 7 shows the number of people posting true or false information over time. It can be seen from Figure 7 that when the safety and material needs are both moderate, the number of people expressing their own opinions is small and the information has two sides. At this time, the information guidance mechanism may have played a role. In other cases, the number of people expressing their own opinions is larger and information is true, which has not changed the original panic buying situation. Therefore, the information review and guidance mechanism is necessary, but people have the right of freedom speech right. As long as they are not spreading rumors, they have the right to express their dissatisfaction. At this time, the intervention effect of the information review and guidance mechanism is not obvious.

### Impact of Official Response on Panic Buying

As the main body responsible for handling public incidents, the government has a huge influence in panic buying and other mass incidents. If the government can report information, dispel rumors, and answer the doubts of the people timely, it can play a positive role. In order to analyze the impact of official response on panic buying, our experiments set the impact parameter on material need λ_1_ = 0.2, *Gov*(*t*) = 1, *t*_*timelyG*_ = 1, and *TR*_*gov*_ = 1.

**Figure 8:**
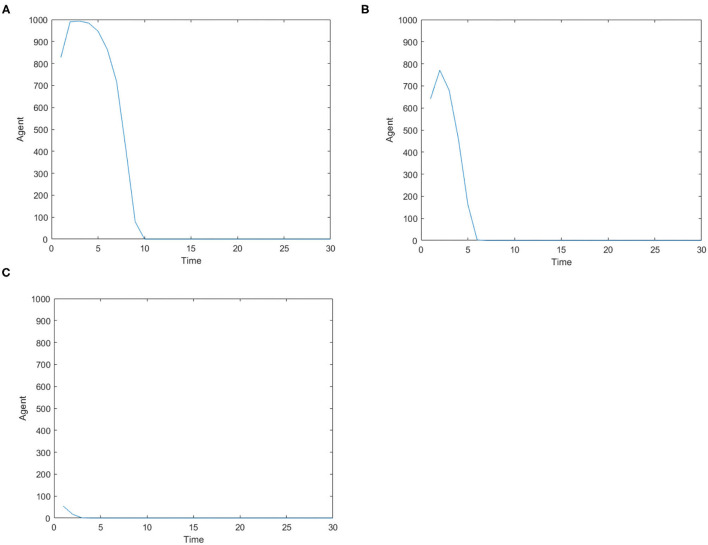
The number of panic buyers over time under different need situations under the official response. **(A)** High safety need, high material need. **(B)** Low safety need, high material need. **(C)** Medium safety need, medium material need.

Figure 8 represents the number of panic buyers over time under different needs situations under the official response. Comparing Figures 2A,C,E, 8A–C, we find that under the official intervention, the number of panic buyers dropped rapidly after reaching the peak, and drops to 0 before time = 1 0. The maximum number of panic buyers reduced from 1,000 to about 800 under the condition of low safety need and high material need. This shows the official response measures curb panic buying immediately.

It is noted that different countries and regions have different levels of trust in the government, which may lead to different levels of intervention in official responses. The following simulates the inhibitory effect of the official response on panic buying when *TR*_*gov*_ = 0.5.

**Figure 9:**
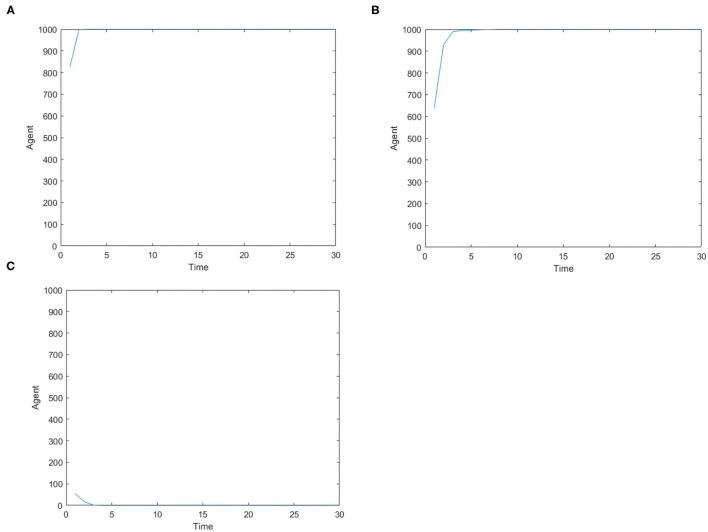
The number of panic buyers over time when the public has low trust in the government. **(A)** High safety need, high material need. **(B)** Low safety need, high material need. **(C)** Medium safety needs, medium material needs.

Figure 9 shows the change in the number of panic buyers over time when the public has low trust in the government. Comparing Figures 8, 9, we see that when people's trust in the government decreases, the inhibitory effect of official responses also decreases. When the material need is high, people's panic buying behavior is not affected by the official response; when the material need is low, the official response can have a certain restraining effect. Therefore, the government must increase people's credibility in order to respond quickly to public incidents and play an important role.

In addition, considering the timeliness of the information, whether the official response is timely may also be a factor that affects the result of the intervention. Set *t*_*timelyG*_ = 3, i.e., when the official response has a certain delay, it has inhibitory effect on panic buying.

**Figure 10:**
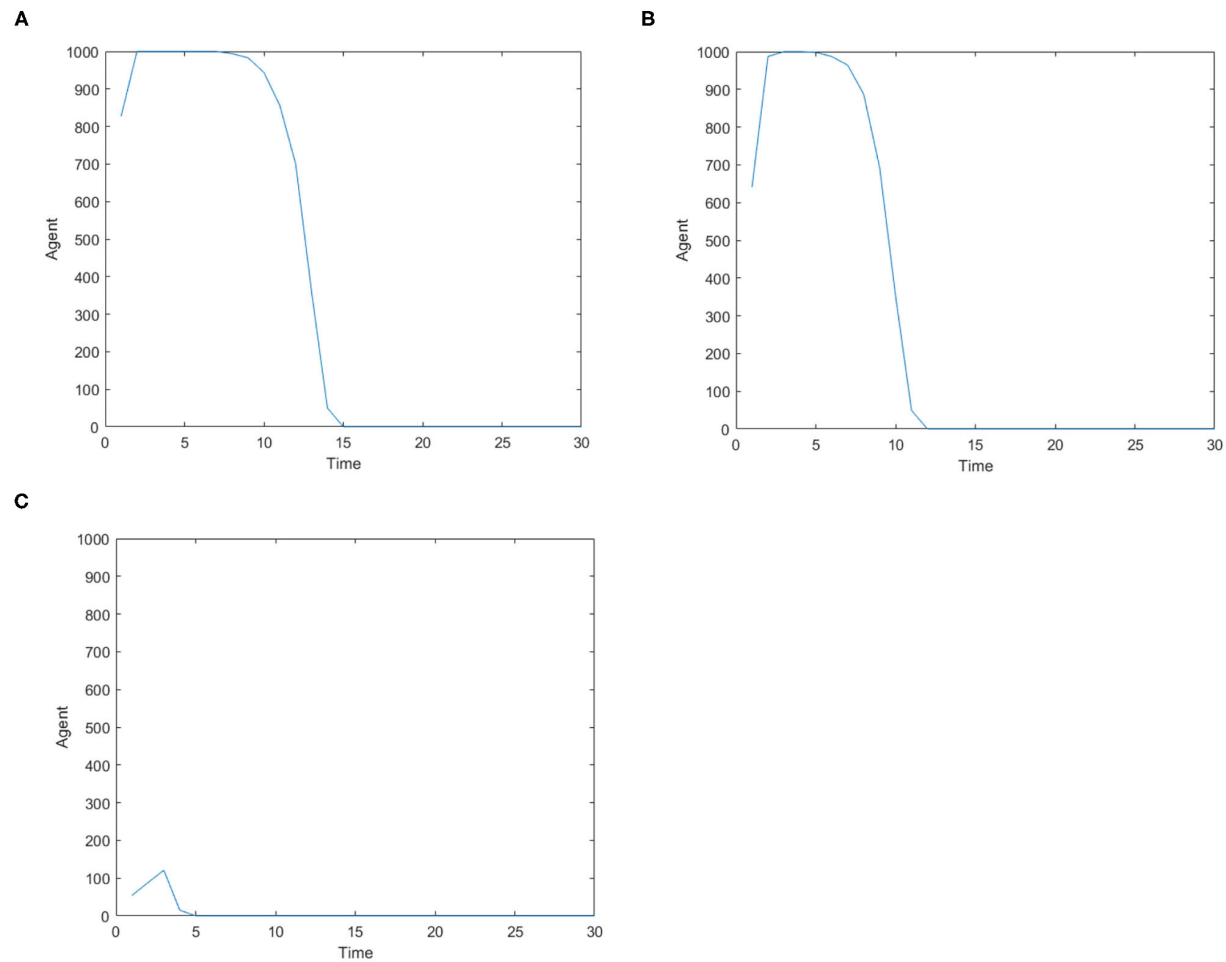
The number of panic buyers over time when there is a delay in the official response. **(A)** High safety need, high material need. **(B)** Low safety need, high material need. **(C)** Medium safety need, medium material need.

Figure 10 shows the number of panic buyers over time when there is a delay in the official response. Comparing Figures 8, 10, we can see that the delay of official response will lengthen the duration of panic buying. In the three cases, the duration of panic buying has increased from time = 10, 6, 3 to time = 15, 12, 5. At the same time, the delay in the official response will also increase the number of panic buyers. The maximum number of panic buyers increased from 800 to 1,000 when the safety need was low and the material need was high, while the maximum number increased from 54 to 121 when safety need and material need are medium. Therefore, relevant agencies should grasp the timeliness of information response and respond in a timely manner.

### Impact of Psychological Counseling on Panic Buying

When people are in a panic, they will be confused and debating with everything. At this time, if there are professionals to guide the people from the perspective of mental health, they may be more relaxed. In order to study the influence of psychological counseling on panic buying, firstly setting λ_2_ = 0.2, *Str*_*PR*_(*t*) = 1, *t*_*timelyP*_ = 1, and *TR*_*ol*_ = 1.

**Figure 11:**
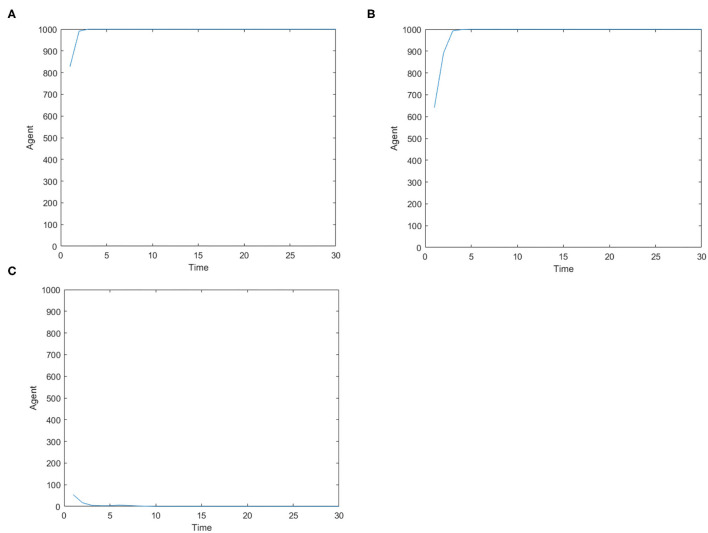
The number of panic buyers under different need conditions under psychological counseling. **(A)** High safety need, high material need. **(B)** Low safety need, high material need. **(C)** Medium safety need, medium material need.

Figure 11 shows the number of panic buyers under different needs conditions with psychological counseling. Comparing Figures 2A,C,E, 11A–C, it can be seen that when the material needs is high, panic buying behavior has not been affected by psychological counseling measures; when the material needs is low, psychological counseling measures still have a certain inhibitory effect.

**Figure 12:**
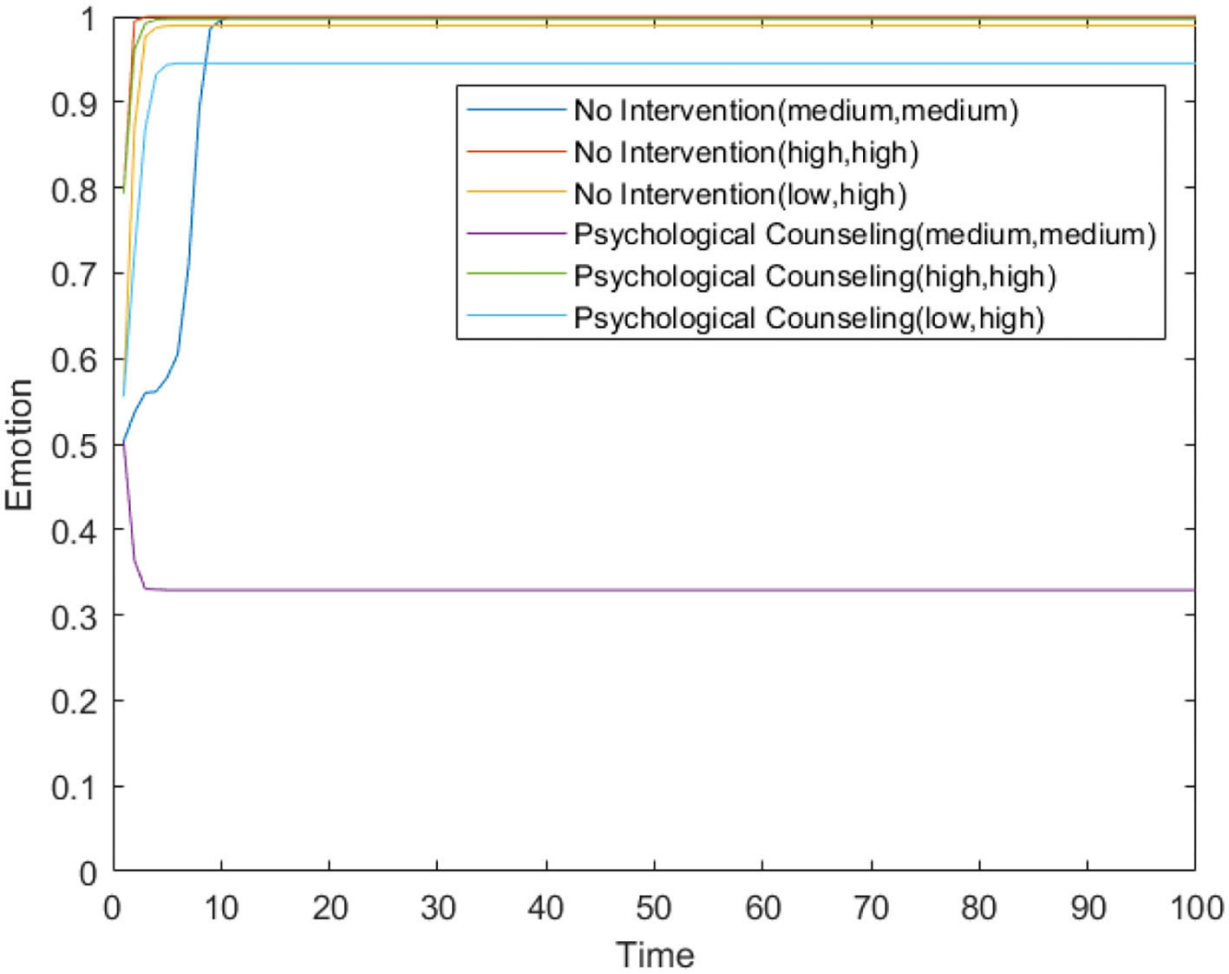
The change of the average panic mood value over time with or without psychological counseling intervention.

Figure 12 compares the change of the average panic mood value over time with or without psychological counseling intervention. It can be seen from Figure 12 that in the case of medium need, psychological counseling measures have a strong regulatory effect on panic emotions, which can affect the occurrence of subsequent panic buying behavior. When the material need is high, the regulatory role of psychological counseling measures is weak and cannot affect the follow-up panic buying. Therefore, psychological counseling has a limited effect on panic buying interventions, and the effect is better when the material needs is not high; and when the material needs is high, it needs to be used in conjunction with other measures.

### Impact of Combined Intervention Measures

The above simulation studies the intervention effect of a single measure. This section explores the combined implementation effect of different intervention measures. Firstly, it simulates the impacts of 4 measures implemented at the same time on panic buying, and discusses the overall intervention effect; secondly, it combines different measures to form 10 combined strategies, analyzes their impacts on panic buying, and then selects the best intervention plan.

**Figure 13:**
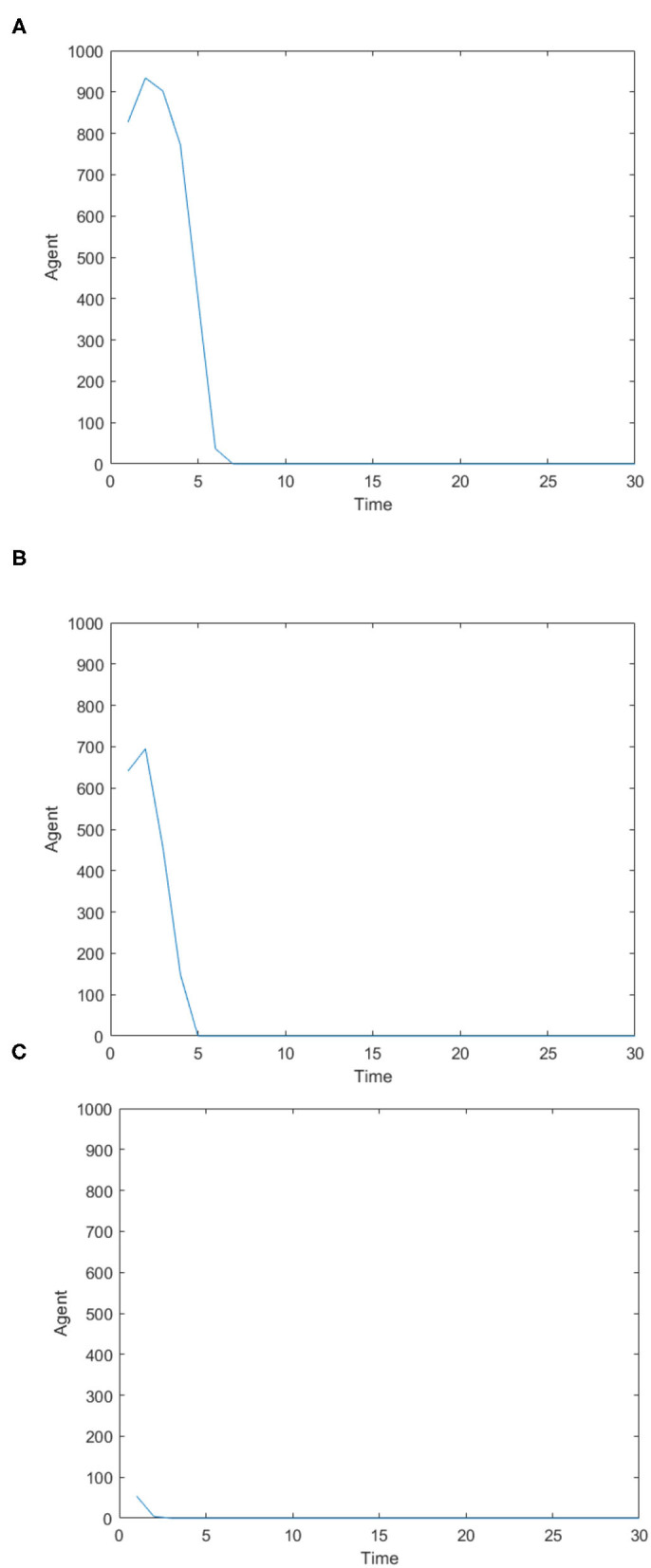
The number of panic buyers over time under different need conditions under the comprehensive plan. **(A)** High safety need, high material need. **(B)** Low safety need, high material need. **(C)** Medium safety need, medium material need.

Figure 13 shows changes in the number of panic buyers over time under different needs conditions under the comprehensive plan. Comparing Figures 2A,C,E, 13A–C, it can be seen that the model proposed in this article has a good effect on panic buying events in different situations, and can effectively curb the spread of panic buying and shorten the duration of panic buying incidents.

From the perspective of a single type of intervention, through the above simulation, it is not difficult to find that the best intervention measure is supply monitoring, and the effective solution to the shortage of supplies in real life is the most effective measure to curb panic buying. The second is the official response, but whose effect is affected by the government's credibility and timeliness. Finally, the information review and guidance mechanism and the psychological counseling have a mediocre intervention effect on panic buying, and they need to be used in conjunction with other measures.

**Figure 14:**
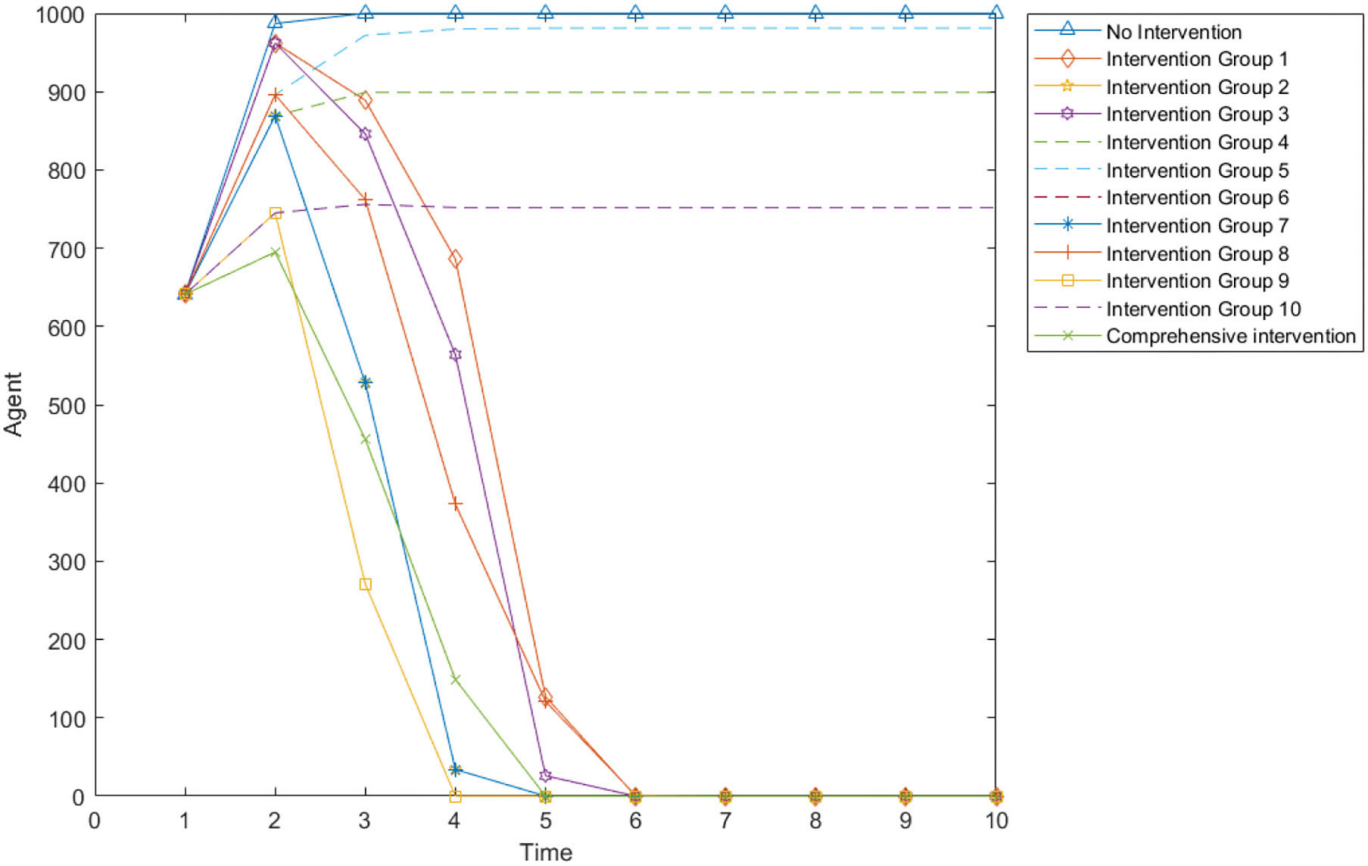
Number of panic buyers over time under different combination.

Ten combination strategies are formed to analyze the intervention effects of different combination strategies. Supply monitoring (referred to as supply in the table below), information review and guidance mechanism (referred to as information in the table below), official response (referred to as official in the table below), and psychological counseling (referred to as psychology in the table below) are combined to form 10 combination strategies. The impact of 10 combination strategies on panic buying in an environment with low security need and high material need is simulated, which is compared with the non-intervention plan. The simulation results are shown in Table 6 and Figure 14.

**Table 6:** Panic buying situation under different combination strategies.

**Plan number**		**Combination type**	**Maximum number of buyers**	**Time to reach the maximum number of buyers**	**Is the panic buying finally stopped**	**Time to stop panic buying**
/	No intervention		1,000	3	No	/
1	Combination strategies	Supply + Information	889	3	Yes	6
2		Supply + Offical	869	2	Yes	5
3		Supply + Psychology	962	2	Yes	6
4		Information + Offical	899	3	No	/
5		Information + Psychology	981	5	No	/
6		Offical + Psychology	756	3	No	/
7		Supply + Information + Offical	869	2	Yes	5
8		Supply + Information + Psychology	896	2	Yes	7
9		Supply + Offical + Psychology	745	2	Yes	4
10		Information + Offical + Psychology	756	3	No	/
/	Comprehensive plan (Supply + Information + Offical + psycholog)		694	2	Yes	5

Table 6 shows the panic buying situation under different combination types, including 12 combinations. Figure 14 presents the data in Table 6 with the manner of a line graph.

Comparing the 10 combinations, it can be found that the 9th group (supply + official + psychology) has the best intervention effect, and the worst is the 5th group (information + psychology). Comparing the 3rd group (Supply + Psychology) and 8th group (Supply + Information + Psychology), it can be seen that although the information review and guidance mechanism has been added, the maximum number of panic buyers at this time has decreased, but the time to end panic buying has increased, indicating that when monitoring and official responses are combined, the information review and guidance mechanism will have a certain counter-effect and extend the time for panic buying. This may be because there is a certain lag in information review and guidance. This measure is to review the information sent by panic buyers, and then guide the information, which has a time difference with the real-time situation. For example, when the first batch of people rushed for purchases, they found that the materials were insufficient and posted relevant information on the Internet, prompting more people to participate in the second batch of rushing purchases. But authorities quickly restocked supplies after the first batch of people bought them, and the second batch of people find that the materials are sufficient. This difference is not because the first batch of people spread misinformation, but because there is a certain time difference between the time when the people released the panic buying information and the actual material replenishment time.

The difference between the 9th group (supply + official + psychology) and the comprehensive plan is that the latter has an additional “information review and guidance mechanism.” Comparing the two situations, it can be found that although the maximum number of buyers in the 9th group is slightly more than that of the comprehensive plan, the stop of panic buying is earlier than the comprehensive plan, which further proves that the “information review and guidance mechanism” will extend the duration of panic buying to a certain extent.

**Figure 15:**
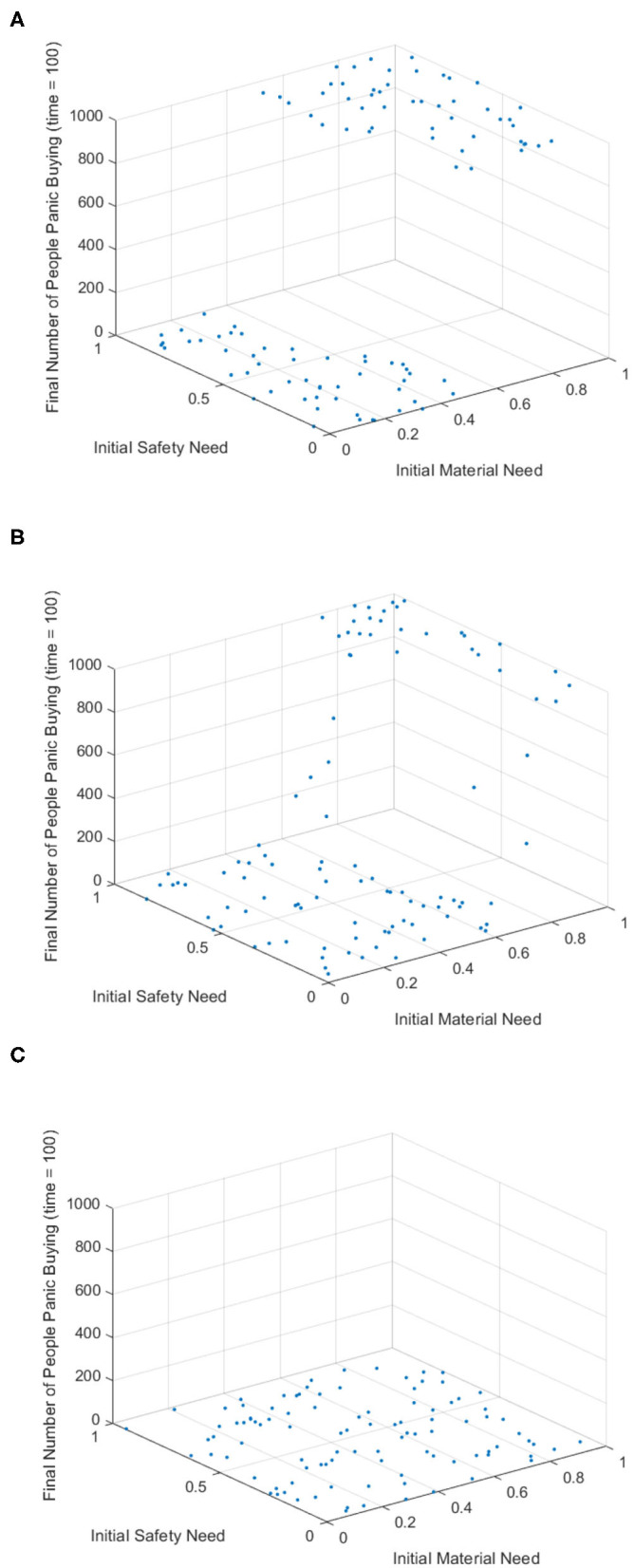
Initial safety needs, initial material needs, and final panic buyers under three intervention scenario. **(A)** No intervention. **(B)** Combined intervention excluding supply monitoring. **(C)** Combined intervention including supply monitoring.

The 4th, 5th, 6th, and 10th groups do not include supply monitoring. Under these 4 combinations, the panic buying did not stop, and the number of panic buyers remained between 700 and 1,000, while the other combinations eventually stopped. In order to analyze whether supply monitoring is a key measure to stop panic buying, the initial safety needs and material needs are randomly set. The comprehensive plan of situation monitoring (supply + information + official + psychology) was carried out with 100 simulation experiments under these three intervention scenarios, and the initial needs value and the final number of people panic buying for each experiment were saved. The result is shown in Figure 15.

Figure 15 shows initial safety needs, initial material needs, and final panic buyers under three intervention scenarios. The x-axis is the initial safety needs, the y-axis is the initial material needs, and the z-axis is the final panic buyers (the number of panic buyers at time = 100), in which each blue dot represents the result of an experiment. From Figure 15A, when there is no intervention, if the initial material needs is high, the final number of buyers will be 1,000, and the panic buying will not stop; if the initial material needs is low, the final number of buyers will be 0, and the panic buying will stop. The number of experiments to stop panic buying and not to stop panic buying is similar. It can be seen from Figure 15B that compared with no intervention, the number of experiments that finally stopped panic buying in this intervention scenario increased, but there were still some cases where panic buying was not stopped. It can be seen from Figure 15C that the effect of this intervention scenario is better, and the number of final buyers is 0, and the number of experiments that have not stopped buying is 0. Therefore, the supply monitoring has played a key role in curbing panic buying.

## Empirical Analysis

Since this article builds a model on the basis of intervention methods under the context of Chinese panic buying incident, in order to verify the effectiveness of the model, this section first selects panic buying incidents that occurred in China for verification. In order to verify the feasibility and applicability of model's performance under different combinations of measures in other countries, the panic buying incident that occurred in the UK is selected for verification.

### Case Study

#### Case 1: Panic Buying Incident in China

December 2019, some hospitals in Wuhan City, Hubei Province, China detected multiple cases of pneumonia of unknown cause initially. Subsequently, COVID-19 spread rapidly around the world. In China, due to the adoption of active public health intervention measures, starting from March 2020, all provinces have resumed work, production and school, and social life has basically returned to normal (48).

However, on January 4, 2021, 127 cases of COVID-19 recurred in Shijiazhuang City, Hebei Province, China. The epidemic reappeared in China, and Shijiazhuang urgently declared a wartime state (49). On January 6, 2021, citizens of Shijiazhuang went to the supermarket to buy daily necessities such as rice, noodles, grain and edible oil (50). From January 7th to January 10th, in order to avoid another panic buying craze, Shijiazhuang City released news related to guaranteeing basic living supplies. For example, 70 supermarkets in Shijiazhuang promised not to increase the price of storage-resistant vegetables (51).

##### Intervention Measures

In order to analyze the effect of intervention measures taken by China during this period, “panic buying in Shijiazhuang” is searched in Baidu, China's largest search engine. Finally, 645 related news from January to February are obtained.

According to the content of the news, combined with the definitions of various measures in the panic buying social intervention measures in Table 3, our research sort out all news according to the type of measures. Since the information review mechanism did not appear in the news, it accounted for 0%; information guidance accounted for 38.9%, official responses accounted for 48%, psychological counseling accounted for 4.5%, and supply monitoring accounted for 8.3%. The various measures are further explored below.

The highest proportion of measures is official response, followed by information guidance.

Since the information guidance mechanism only has a setting of presence or absence in the model, an information guidance mechanism is set in the case simulation.

Official response and psychological counseling belong to the information spread of the government and opinion leaders. The earliest news of the panic buying event in Shijiazhuang released on January 6, 2021 and was regarded as the beginning of the event. In terms of timeliness, the timeliness of such measures is measured by calculating the average time difference between the release time of a certain type of measure at this stage and the earliest release time. It can be concluded that the average time difference between official response measures and psychological counseling measures is 1.41 days and 1 day, respectively, indicating that both the government and opinion leaders have expressed their opinions in a timely manner. Since the number of days in the simulation experiment can only be an integer, our experiments set *t*_*timelyG*_ = 2, and *t*_*timelyP*_ = 1. In terms of information intensity, the proportion of official response measures is about 10 times that of psychological counseling measures. Therefore, *Gov*(*t*) = 10, *Str*_*PR*_(*t*) = 1. In terms of public trust, according to a recent survey conducted by an independent agency in Singapore, China ranks first in the evaluation of local governments by citizens of 23 countries/regions (52). Therefore, our experiments set *TR*_*gov*_ = 1, and *TR*_*ol*_ = 0.8.

Supply monitoring is an offline measure. By analyzing the text content of relevant news, it is found that the China Market Supervision Administration has inspected the sales of food and drugs, agricultural and sideline products, and daily consumer goods in its jurisdiction to ensure sufficient supplies. During this period, two cases were investigated and prosecuted. Therefore, the supply monitoring measures in this case are better implemented, with the setting *Q*_*move*_(*t*) = 1.

##### Panic Buying Behavior Measurement

The public is the main body of panic buying behavior. Since the specific number of panic buying offline is difficult to measure, the discussion of relevant topics on Weibo is used to measure netizens' panic buying behavior. Weibo is China's leading social media company, with more than 511 million monthly active users. The user comment data accumulated on the platform can objectively reflect the public's views on various events.

Supplementary Figure 4 presents topic index of #panic buying in Shijiangzhuang#. The discuss trend represents the change in the number of netizens who post related Weibo content, comment, like, and repost other related Weibo content, and the number of original creators. It represents the change in the number of related Weibo content posted by netizens on their own, and to a certain extent can reflect the enthusiasm of Shijiazhuang panic buyers. There are two crests in the two trend graphs (represented by the red dots in the figure), the big crest on January 6 and the small crest on January 9, which shows that the residents of Shijiazhuang have shown a two-stage change in panic buying. Netizens' enthusiasm for panic buying was very high on the 6th, but on January 9th, the enthusiasm of netizens was very low.

#### Case 2: Panic Buying Incident in the UK

In March 2020, affected by the spread of COVID-19, the United Kingdom set off a trend of hoarding living supplies. There was a great number of panic buyers in London for buying toilet article, hand sanitizer, canned food and other items. In December 2020, due to the emergence of variants of COVID-19, the United Kingdom announced the highest level of “level 4” blockade restrictions in London and the southeastern part of the United Kingdom. In addition, the Brexit has also hindered the import of goods to a certain extent. Some people worried about the shortage of goods, which turned into a panic buying frenzy.

##### Intervention Measures

In order to analyze the differences in intervention measures taken by the UK in two different periods, relevant news with #UK panic buying# and #London panic buying# as keywords are collected in the most authoritative news media website BBC News in the UK. Finally, 456 news from March to April 2020 and 431 news from December 2020 to January 2021 are obtained.

According to the content of the news, combined with the definitions of various measures in the panic buying social intervention measures in Table 3, all news are sorted according to the types of measures, and the results are shown in Table 7. It can be seen from Table 7 that since the information review mechanism did not appear in the news, it accounted for 0%; March was better than December in psychological counseling and supply monitoring; December was better than March in information guidance. There is no significant difference between the two in official response.

**Table 7:** Proportion of intervention measures at different stages.

**Time**	**March %**	**December %**
Information guidance-1	16.8	40.8
Information review-2	0.0	0.0
Official response-3	39.4	38.8
Psychological conselling-4	12.4	5.1
Supply monitoring-5	31.4	15.3

The following further explores the various measures, and the research method is similar to Case 1.

In the actual case, the proportion of information guidance measures in December is much higher than that in March. Therefore, in the case simulation, there is no information guidance mechanism in March and there is an information guidance mechanism in December.

In terms of the timeliness of official responses and psychological counseling measures, the timeliness of such measures is measured by calculating the average time difference between the release time of a certain type of measures at this stage and the earliest release time. The earliest news release time in March is March 2nd, and the earliest news release time in December is December 10th, which is the beginning of the event. It can be concluded that the average time difference between psychological counseling measures in March and December is 16 and 25 days, respectively, and the average time difference between official response measures is 15 days. In terms of information intensity, as shown in Table 7, the proportion of psychological counseling measures in March was about twice that of December, and the proportion of official response measures was similar. Therefore, in order to facilitate simulation, our experiments set *t*_*timelyP*_ is 1, *t*_*timelyG*_ is 1, *Gov*(*t*) is 1, *t*_*timelyP*_ is 2 in December, *Str*_*PR*_(*t*) is 0.5, *t*_*timelyG*_ is 1, *Gov*(*t*) is 1. In terms of public trust, according to a recent survey conducted by an independent agency in Singapore, the UK ranks only the 15th in the evaluation of local governments by citizens of 23 countries/regions (34), which may be related to the failure of British herd immunity. Therefore, our experiments set *TR*_*gov*_ is 0.5, and *TR*_*ol*_ is 0.8.

Supply monitoring is an offline measure. By analyzing the text content of relevant news, it is found that in March and December, all major supermarkets in the UK used measures such as purchase restrictions and quotas to ensure adequate supply of materials, but the difference is: the UK also has not affected by Brexit in March. However, the material transfer became difficult after being affected by Brexit in December. Therefore, setting *Q*_*move*_(*t*) is 1 in March and *Q*_*move*_(*t*) is 0.5 in December.

##### Measurement of Panic Buying Behavior

Since it is difficult to measure the specific number of persons for panic buying offline, the people's emotional response to panic buying under these two different periods and different intervention environments is used as an indicator to measure the occurrence of panic buying. Use #UK panic buying# and #London panic buying# as keywords on Twitter to get relevant tweets and comments from March to April 2020 and December 2020 to January 2021. In the end, a total of 247 tweets and 15,656 comments were crawled. The tweets unrelated to panic buying are cleared, and then the data is preprocessed. Finally, a total of 157 tweets and 8,543 comments are obtained.

Emotion analysis on the comment data is conducted. Use the emotion dictionary on CNKI (Chinese National Knowledge Infrastructure) to perform emotion analysis based on Python. Through the scoring of emotional words, degree words, and emoticons, the emotion score of each comment is finally obtained. After statistical collation, the summary is shown in Table 8. It can be seen from Table 8 that the proportion of people's negative emotion on material topics in December was higher than that in March.

**Table 8:** Emotion analysis in case 2.

**Statistical items**	**March**	**December**
	**Material topic**	**Material topic**
Number of positive comments	1,422	1,912
Number of negative comments	647	901
Number of neutral comments	1,561	2,100
Proportion of negative emotions	17.8%	18.3%

### Case Simulation

The following simulates the event based on the model mentioned in this article. Due to the large amount of case data, considering comprehensive visualization, the network scale of the simulation is set to 1,000. Since the incident was originally caused by external epidemic news, it is necessary to set the initial value of individual needs.

The initial value of the individual material needs is measured by the number of crawled material reviews. Therefore, in Case 1, the initial value of material needs *M*_0_ is set to 0.5, and in Case 2, the initial value of material needs *M*_0_ is 0.6 in March, and 0.8 in December. The initial value of the individual's safety needs is measured by the number of new deaths per day. On January 6, 2021, there were no new deaths in China, so the initial value of safety needs *S*_0_ in Case 1 was set to 0. On March 25, 2020, the number of new deaths in the United Kingdom was 148, and on December 22, 2020 it was 215, so the initial value of safety needs in March in Case 2 is set to 0.3, and the initial value of safety needs in December is set to 0.6. The parameter setting of intervention measures has been introduced in detail in the section “Intervention measures combing.” The other parameters are set as follows: the individual's conformity degree *Con*(*i*) obeys the normal distribution of *N* ~ (0.5, 0.15) and is mapped to [0, 1], indicating that the majority of the individual's conformity degree is medium. The weight of material needs (physiological needs) in individual needs α is 0.6, and the weight of safety needs in individual needs β is 0.4; the people's own knowledge of their own material needs parameters μ is 0.2; the supply and demand threshold *d*_*SD*_ is 0.6, and the panic buying threshold *d*_*A*_ is 0.5. The influence of official response parameter on material need λ_1_ is 0.1, and the influence of psychological counseling on panic emotion λ_2_ is 0.1.

First, simulate the panic buying process of Case 1, and the result is shown in Supplementary Figure 5.

Supplementary Figure 5 shows the number of panic buyers over time in the simulation case 1. It can be seen from Supplementary Figure 5 that in the case of intervention, the number of panic buyers decreased sharply after the initial increase, and decreased to 0 when Time = 4, indicating the effect of intervention measures on panic buying in this case is fast and efficient. This is similar to the real case in Supplementary Figure 4. The netizens' enthusiasm for panic buying was high at first, but dropped rapidly after just 3 days, which verified the effectiveness of the model.

The panic buying situation of case 2 is simulated, and the result is shown in Supplementary Figure 6.

Supplementary Figure 6 shows the number of panic buyers over time in the simulation case 2. It can be seen from Supplementary Figure 6 that the intervention measures in March have played a significant role. Compared with the situation without intervention, the number of panic buying has dropped rapidly in a short period of time, and there is no panic buying around Time = 10; while in December, panic buying measures did not play a significant role, similar to the situation without intervention, the number of panic buying continued to remain high. The reasons for the difference between March and December may be: (1) the individual's initial needs are different. Compared with December, the basic material needs and safety needs in March are lower. Therefore, people's panic level is lower and their desire to purchase materials is also lower. From Supplementary Figure 6, we can see that in December when Time = 1, almost everyone participated in the panic buying. (2) The intervention measures adopted in the two stages are different. Through simulation experiments, the best intervention effect is the monitoring of the supply situation. In December, due to the Brexit problem, the mobilization of materials was insufficient, and the volume of goods was larger than that in March. Although there is an information guidance mechanism in December, this mechanism needs to be used in conjunction with other measures, and its own intervention effect is mediocre. As a result, the intervention measures in December did not play a significant role.

According to the results of emotion analysis of user reviews, among the comments on supplies, the negative comments in December are higher than those in March, indicating that the public's reaction to panic buying is more intense at this time. Even if the parties take intervention measures, the public's panic cannot be smoothed. This realistic result is consistent with the case simulation result in Supplementary Figure 6.

In summary, the case simulation in this section verifies that the model can simulate panic buying under different intervention plans with flexible manners, which is feasible in real-world applications. It also verifies that the model can be applied to panic buying in different countries and is applicable to the general.

## Conclusions

COVID-19, as an epidemic, has been studied by a large number of scholars from the perspective of medicine. For example, Sung et al. (53) investigated the occurrence of burnout, acute stress disorder, anxiety disorder and depression among medical service providers in the third month of COVID-19 pandemic. Rashidzadeh et al. (54) explored the progress of nano materials in COVID-19 prevention, diagnosis and treatment. Manal (55) explored the impact of the 2019 coronavirus epidemic on the mental health status of primary health care institutions in Dubai. These articles provide a sociological perspective to explore the panic buying behavior under the background of COVID-19. Based on the analysis of the real social intervention measures and the causes of panic buying behavior, our study creatively constructs the social intervention mechanism of panic buying behavior under the sudden epidemic situation and analyzed the role of 5 kinds of social intervention measures in panic buying from the perspectives of online and offline channels, government, social groups and other subjects. Then, through simulation experiments, we explore the impact of single measures and combined strategies on panic buying. Finally, the feasibility and universality of the model are verified by examples. It expands the research dimensions of social intervention mechanism and provides guidance and suggestions for crisis management under public health emergencies.

The following conclusions are obtained through simulation experiments:

1. The best effect single measure is supply monitoring. The size of the material adjustment has an important impact on alleviating panic buying. When monitoring the supply situation, relevant departments need to pay attention to the demand changes of material quantity at different times, and dynamically adjust the material adjustment, so as to alleviate the panic buying phenomenon faster. The official response to the intervention can have an immediate inhibitory effect, but lack of credibility and failure to respond in time will affect the effect of intervention. While the intervention effect of psychological counseling is limited, and it needs to be used in conjunction with other measures when the need for materials is strong.
2. The most effective combination strategy is “supply monitoring + official response + psychological counseling,” and the worst is “information review and guidance + psychological counseling;” supply monitoring is a key measure to curb panic buying. Also, “information review and guidance” will play a certain counter-effect in the combined strategy, which may lead to prolonged buying time.

However, this article still has the following shortcomings, which need further study:

1. Some quantitative parameters of intervention measures cannot be accurately observed in the real world (56), which makes the data in the empirical part of the case relatively ideal.
2. From the perspective of the life cycle of panic buying behavior, since this article focuses on the intervention mechanism of panic buying, it mainly conducts the study of the formation process of panic buying, yet it does not consider the influence of individual forgetting mechanism on the disappearance of panic buying behavior (57).

## Data Availability Statement

The data used to support the findings of this study are available from the corresponding author upon request.

## Author Contributions

PF and TC described the proposed framework and wrote the whole manuscript. BJ implemented the simulation experiments. JY and GC revised the manuscript. All authors contributed to the article and approved the submitted version.

## Funding

This research was supported by the National Social Science Foundation of China (Grant No. 18BGL101), the Zhejiang Provincial Natural Science Foundation of China (Grant No. LY22G010003), the Project of China (Hangzhou) Cross-border E-commerce College (No.2021KXYJ03), and as well as the Characteristic and Preponderant Discipline of Key Construction Universities in Zhejiang Province (Zhejiang Gongshang University-Statistics).

## Conflict of Interest

The authors declare that the research was conducted in the absence of any commercial or financial relationships that could be construed as a potential conflict of interest.

## Publisher's Note

All claims expressed in this article are solely those of the authors and do not necessarily represent those of their affiliated organizations, or those of the publisher, the editors and the reviewers. Any product that may be evaluated in this article, or claim that may be made by its manufacturer, is not guaranteed or endorsed by the publisher.

## Supplementary Material

Supplementary Figure 1 The relation between the number of monthly news related to different types of measures and panic buying events.

Supplementary Figure 2 Framework.

Supplementary Figure 3 The idea of model construction.

Supplementary Figure 4 Topic index of #panic buying in Shijiangzhuang#. **(A)** Discuss trend. **(B)** Original trend.

Supplementary Figure 5 The number of panic buyers over time in the simulation case 1.

Supplementary Figure 6 The number of panic buyer over time in the simulation case 2. **(A)** March. **(B)** December.
