# Supplementary figures and images for: Identifying a New Social Intervention Model of Panic Buying Under Sudden Epidemic
Source: Front Public Health. 2022 Mar 11;10:842904. doi: 10.3389/fpubh.2022.842904 (PMC8963413; doi:10.3389/fpubh.2022.842904)

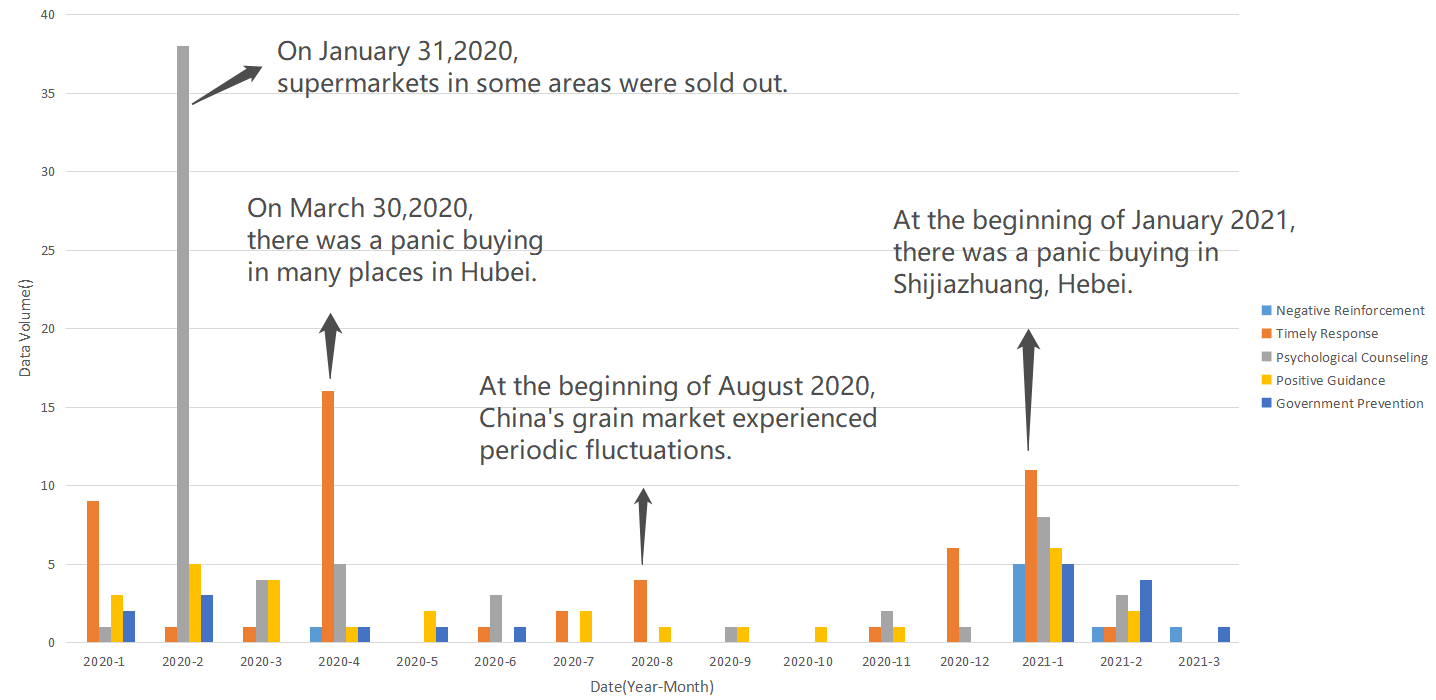

Supplement: Supplementary Figure 1 — The relation between the number of monthly news related to different types of measures and panic buying events. [file Presentation_1.zip › figures/Figure1.png]

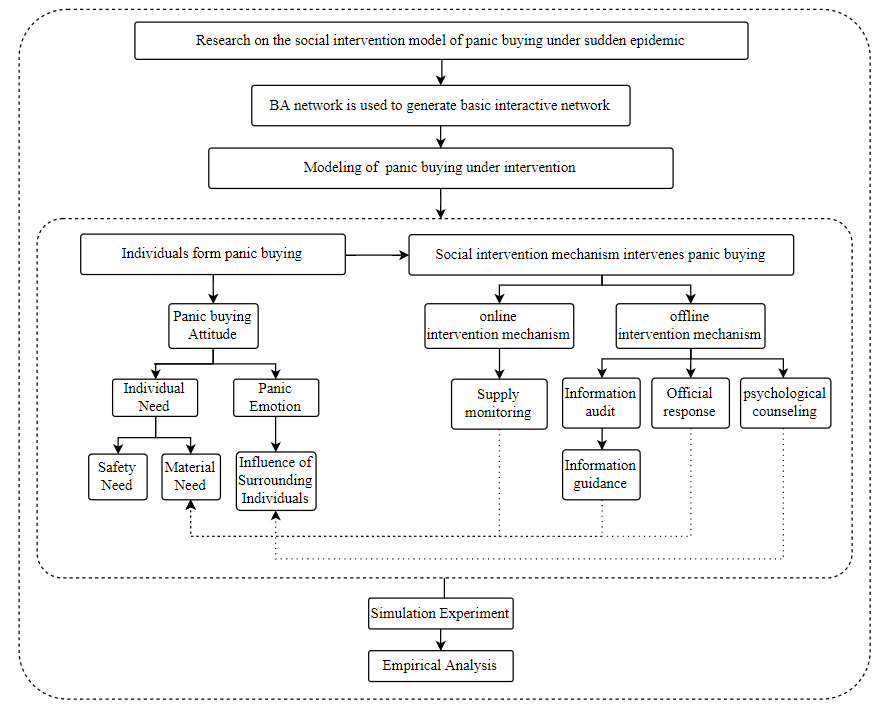

Supplement: Supplementary Figure 1 — The relation between the number of monthly news related to different types of measures and panic buying events. [file Presentation_1.zip › figures/Figure2.png]

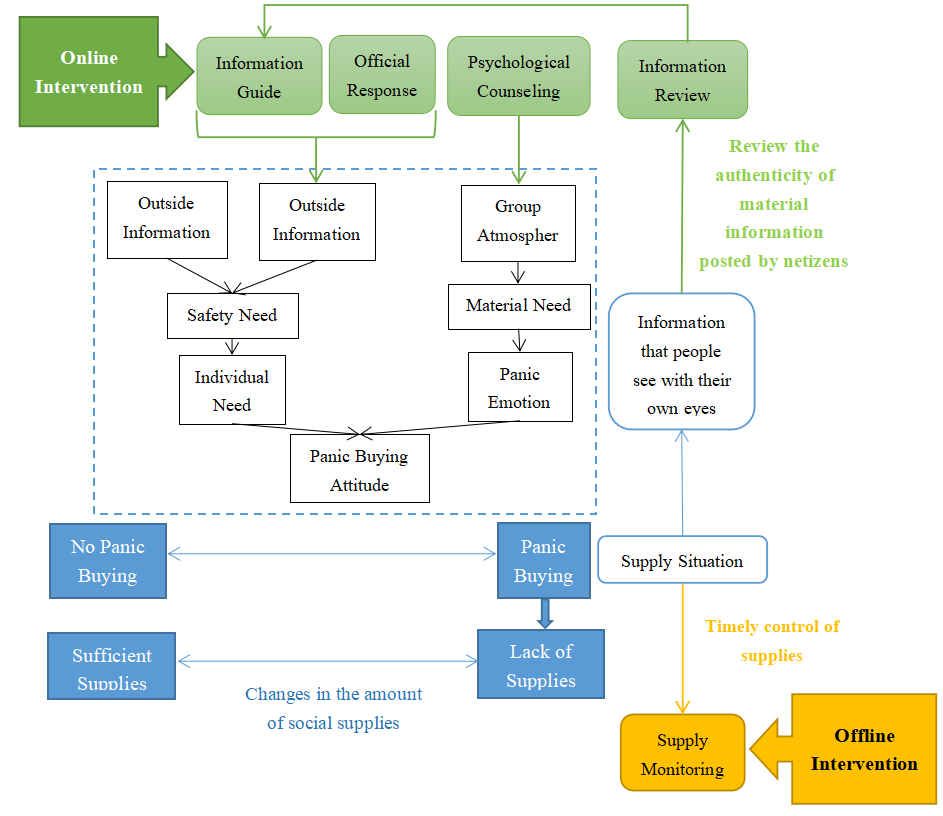

Supplement: Supplementary Figure 1 — The relation between the number of monthly news related to different types of measures and panic buying events. [file Presentation_1.zip › figures/Figure3.png]

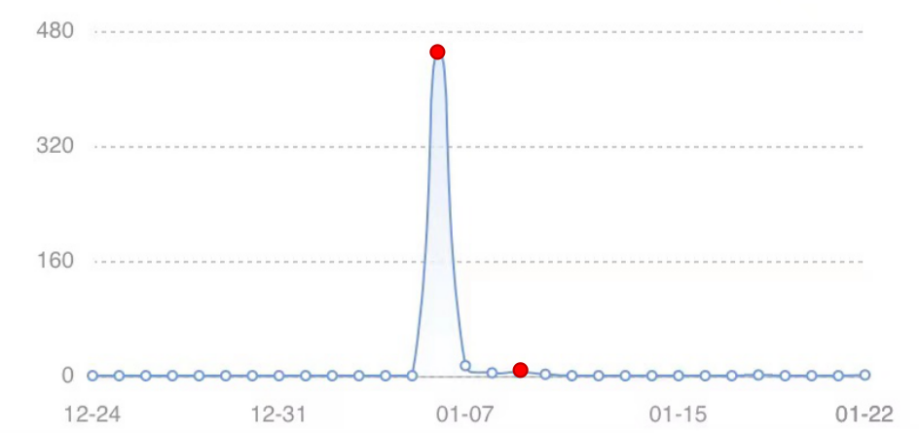

Supplement: Supplementary Figure 1 — The relation between the number of monthly news related to different types of measures and panic buying events. [file Presentation_1.zip › figures/Figure4(a).png]

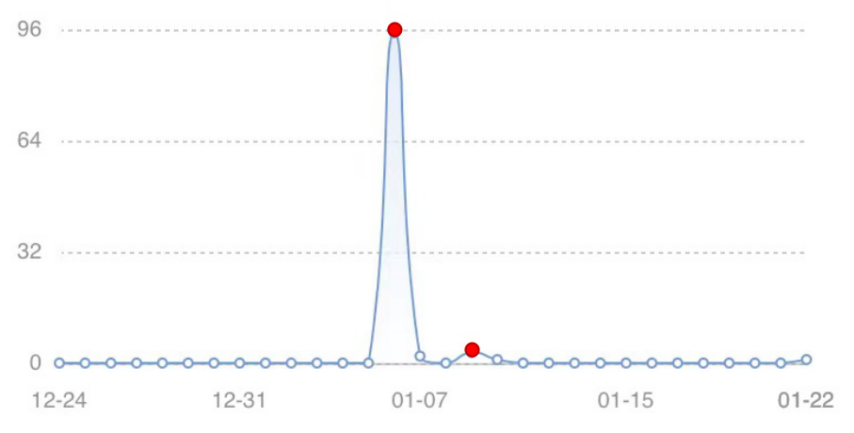

Supplement: Supplementary Figure 1 — The relation between the number of monthly news related to different types of measures and panic buying events. [file Presentation_1.zip › figures/Figure4(b).png]

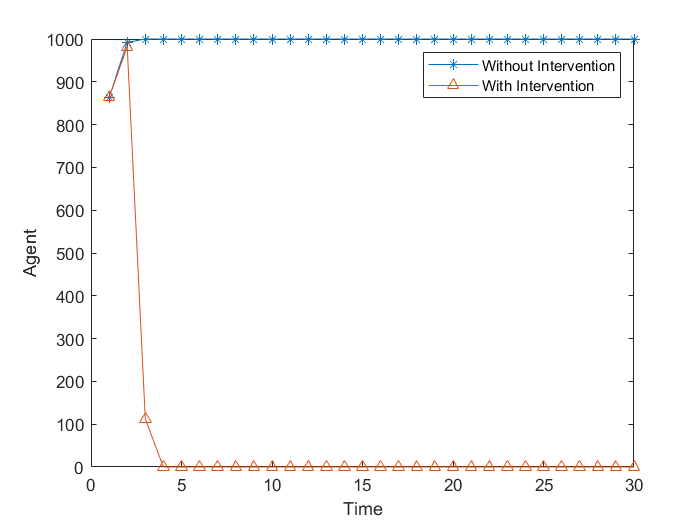

Supplement: Supplementary Figure 1 — The relation between the number of monthly news related to different types of measures and panic buying events. [file Presentation_1.zip › figures/Figure5.png]

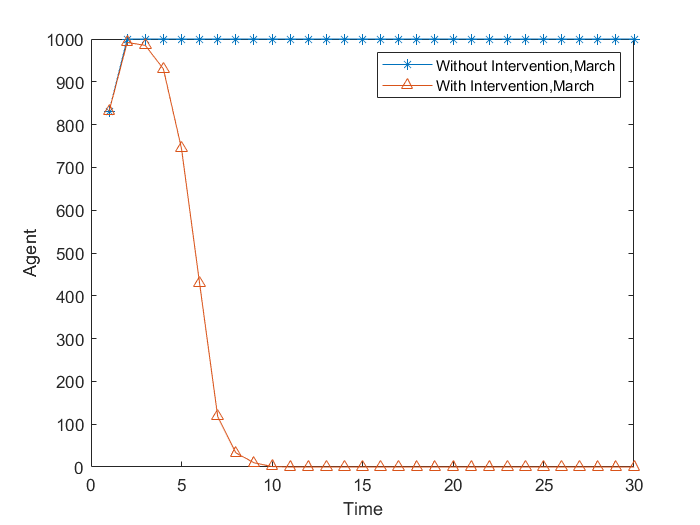

Supplement: Supplementary Figure 1 — The relation between the number of monthly news related to different types of measures and panic buying events. [file Presentation_1.zip › figures/Figure6(a).png]

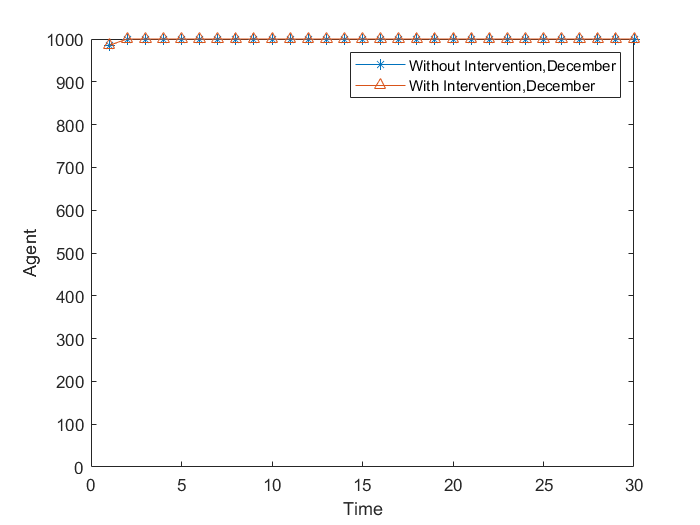

Supplement: Supplementary Figure 1 — The relation between the number of monthly news related to different types of measures and panic buying events. [file Presentation_1.zip › figures/Figure6(b).png]
